# Changes in Dietary Behaviours during the COVID-19 Outbreak Confinement in the Spanish COVIDiet Study

**DOI:** 10.3390/nu12061730

**Published:** 2020-06-10

**Authors:** Celia Rodríguez-Pérez, Esther Molina-Montes, Vito Verardo, Reyes Artacho, Belén García-Villanova, Eduardo Jesús Guerra-Hernández, María Dolores Ruíz-López

**Affiliations:** 1 Department of Nutrition and Food Science, University of Granada, Campus of Cartuja, 18071 Granada, Spain; vitoverardo@ugr.es (V.V.); rartacho@ugr.es (R.A.); belenv@ugr.es (B.G.-V.); ejguerra@ugr.es (E.J.G.-H.); mdruiz@ugr.es (M.D.R.-L.); 2 Institute of Nutrition and Food Technology (INYTA) ‘José Mataix’, Biomedical Research Centre, University of Granada, Avenida del Conocimiento s/n, E-18071 Granada, Spain; 3 Instituto de Investigación Biosanitaria ibs. GRANADA, University Hospital of Granada/University of Granada, 18011 Granada, Spain

**Keywords:** dietary behaviours, COVID-19, confinement, Mediterranean diet, olive oil, vegetables, fruits, legumes, fried foods, snacking

## Abstract

The aim of this study was to evaluate whether dietary behaviours of the Spanish adult population were changed during the COVID-19 outbreak confinement. For that purpose, an online questionnaire, based on 44 items including socio-demographic data, Mediterranean diet (MedDiet) Adherence Screener (MEDAS) as a reference of a healthy diet, processed foods intake, changes in their usual food choices and weight gain was distributed using social media and snowball sampling. A total of 7514 participants (37% aged below 35 years, 70.6% female, 77.9% university-level education or higher) from all the Spanish territory completed the questionnaire. Results outlined healthier dietary behaviours during the confinement when compared to previous habits. Overall, the MEDAS score (ranging from 0 to 14, whereby higher a scoring reflects greater adherence to the MedDiet) increased significantly from 6.53 ± 2 to 7.34 ± 1.93 during the confinement. Multivariate logistic regression models, adjusted for age, gender, region and other variables, showed a statistically significant higher likelihood of changing the adherence to the MedDiet (towards an increase in adherence) in those persons who decreased the intake of fried foods, snacks, fast foods, red meat, pastries or sweet beverages, but increased MedDiet-related foods such as olive oil, vegetables, fruits or legumes during the confinement. COVID-19 confinement in Spain has led to the adoption of healthier dietary habits/behaviours in the studied population, as reflected by a higher adherence to the MedDiet. This improvement, if sustained in the long-term, could have a positive impact on the prevention of chronic diseases and COVID-19-related complications.

## 1. Introduction

The 2019 coronavirus (COVID-19) pandemic, caused by SARS-CoV-2, has expanded from Wuhan, the capital city of Hubei Province in China to a growing number of countries [1]. To date, more than 1,333,711 cases of coronavirus have been reported in Europe and concretely, 207,634 cases came from Spain, according to the latest Coronavirus disease 2019 situation report [2]. Due to the gravity of circumstances, the ‘State of alarm’ was declared in Spain on 14th March 2020 [3]. Since then, the Spanish population was firstly ordered to stay at home for two weeks that were later extended by another two weeks to halt the spread of coronavirus, paying more attention to vulnerable populations including persons with underlying medical conditions or with a compromised immune system from a medical condition or treatment, health care providers, or older adults [4]. Under that situation, people were allowed to leave home to buy essentials from supermarkets and pharmacies, go to work in those cases in which work in-person was essential (pharmacies, healthcare, supermarkets, etc.) while maintaining social distance.

This implementation firstly caused panic buying and disruptions to food supply leading to shortages of some staple foods in most supermarkets and grocery stores. However, after the first four days, most companies ensured sufficient food supply during this period of confinement in view of COVID-19. Despite that the situation seemed to be normalized, not all food and food products were easily available at the supermarkets, making senior citizens even more vulnerable.

Against this emerging and rapidly evolving situation, and independently of the availability/non-availability of food products, people should have had more time for cooking and for organizing their meals. But also, people had more time for watching television (TV) and even to get bored. Watching TV has been associated with snacking frequency, especially energy-dense snacks, fast foods or soda beverages [5,6]. Mittal et al. [7] concluded that snacking while watching television is associated with a promotion of over-consumption on a later meal. On its behalf, boredom has been suggested to be a predictor of eating behaviours [8].

The restriction to stay at home with children in some cases, teleworking and avoiding to be in close contact with other people could also cause stress and anxiety throughout the population [9]. In this regard, further than considering emotional eating, some studies have associated the food choice with psychological parameters, such as anxiety [10,11]. Additionally, the confinement considerably limits physical activity, which could increase the emotional imbalance due to the ‘alarm situation’. Referring to the elderly, social isolation could lead to depression, cognitive dysfunction, disability, higher cardiovascular disease (CVD) risk and even increased mortality, as recently reported by Morley and Vellas [12].

Thus, changes in dietary and physical activity habits as a consequence of COVID-19 confinement were expected. In fact, the Spanish Ministry of Agriculture, Fisheries and Food reported an about 30% increment in food purchases during the week before the confinement started with respect to the same date last year [13]. To support the immune system under situations like this, the establishment of healthy dietary habits is crucial. In this regard, the European Federation of the Association of Dietitians (EFAD) published COVID-19 information for nutritional support [14]. Furthermore, the World Health Organization (WHO) also offered several food and nutritional tips during self-quarantine [15], while the Mediterranean diet (MedDiet) as a healthy nutritional pattern to be followed in quarantine has been recommended [16]. However, so far, no data with respect to the real dietary habits of the population are available. Therefore, the main objective of this study was to examine whether the COVID-19 outbreak confinement has influenced dietary habits among the Spanish adult population. For that purpose, a self-administered web-based questionnaire with questions aimed at assessing the dietary behaviours of the adult Spanish population during the COVID-19 confinement and before it started, was distributed using social media and snowball sampling.

## 2. Materials and Methods 

### 2.1. Study Design and Participants

A cross-sectional study (COVIDiet) was carried out among Spanish adults who were encouraged to participate in the present study without any exclusion criteria further than the age (>18 years old). Participation in the online questionnaire was entirely voluntary and anonymous. The study was conducted in agreement with the Declaration of Helsinki, and all data were collected anonymously and recorded according to the Spanish Organic Law of Personal Data Protection (LOPD) 15/1999. Since the questionnaire is anonymous and no personal data are collected, no informed written consent was requested. However, participants were informed about the objective of the research and they were asked for permission to use and publish the data from the study before starting the questionnaire. This study was approved by the Research Ethics Committee of the University of Granada (1526/CEIH/2020). The questionnaire was open from March 20, concretely one week after the Spanish COVID-19 outbreak confinement started. Data from the 3 first weeks of confinement were collected.

### 2.2. Instruments and Variables

A questionnaire containing 44 items (Supplementary Table S1) was designed for the assessment of data about consumption frequency of selected foods (mainly related with the MedDiet), general food habits and socio-demographic characteristics. In particular, the questionnaire was self-administered and divided into three main sections distributed as follows. Socio-demographic information items i.e., sex, age, place of residence, country, dependent children, and level of studies were included. Furthermore, the weight was requested. Fourteen items with reference to the MedDiet pattern based on the validated PREDIMED MedDiet Adherence Screener (MEDAS) [17], were incorporated in the second section. Those first 14 items were based on a multiple choice close ended question that, together with the way of evaluation, have been described elsewhere [18]. Briefly, one point was scored when participants selected olive oil for cooking, daily consumption of four or more tablespoons of olive oil, white meat vs. red meat, two or more servings of vegetables, three or more pieces of fruit, less than one serving of red meat, hamburgers, sausages or deli meats, less than one serving of carbonated or sugary drinks, weekly intake of seven or more glasses of wine, three or more servings of legumes, three or more servings of fish/seafood, three or more servings of nuts, less than two servings of non-homemade pastries, white meat such as turkey or chicken preference instead red meat and two or more dishes seasoned with tomato, garlic, onion or leeks and sautéed with olive oil (sofrito). Hereby, participants provided information on compliance with every item and the overall MedDiet pattern, both during the COVID-19 confinement and before its entry into force. For the latter, participants were asked in every item whether they had made any actual change due to the current situation. 

Using this information, the adherence to the MedDiet before the COVID-19 confinement was assessed assuming the following: one point was assigned if consumption was maintained as usual in those participants who selected the highest intake for foods that are characteristic of the MedDiet such as olive oil, fruits, vegetables, fruits, pulses or fish/seafood. On the contrary, 0 points were assigned to those participants that maintained their intake as usual (highest intake) non-characteristic MedDiet foods i.e., red meats, hamburgers, sausages or deli meats, sugary beverages or commercial (non-homemade) pastries. Higher scores indicate greater adherence to the MedDiet with a total score ranging from 0 to 14.

For both MedDiet assessments, before and during the confinement, the final score ranged from 0 to 14, with <5, 6 to 8 and >9 points indicating low, medium or high adherence to the MedDiet, respectively. 

Participants were also asked to answer 21 in-house items aimed at investigating changes in their usual dietary habits during the confinement, i.e., way and frequency of cooking, snacking, alcohol intake or type of oil employed for frying, among others. In this case, all questions were also designed to know if participants increased, decreased or maintained their habits during the COVID-19 outbreak confinement. Additionally, participants were also asked whether they perceived that their physical activity and body weight had changed since the confinement started by means of two questions. Duration of confinement was accounted for, both, days and weeks since the study started (in the second week of confinement) until the end of the survey.

### 2.3. Procedure

To try to cover the whole Spanish territory and to reach the greatest number of persons through mobile phones, tablets and computers, the questionnaire was created using the Google Forms tool and was distributed using instant messaging apps e.g., WhatsApp, social media such as Facebook and Twitter, social networking sites such as LinkedIn and ResearchGate and emails through snowball sampling. 

### 2.4. Statistical Analyses

Descriptive statistics for all the collected variables were derived by levels of adherence to the MedDiet and by sex, age, level of education and region. Student’s t-test or Kruskal-Wallis test (for continuous normal or non-normal distributed data, respectively), and Chi-squared tests (for categorical data) were used to evaluate differences in means or proportions by these variables across the strata. Box-plots were also used to evaluate further the distribution of the variable on adherence to the MedDiet by the aforementioned subgroups. 

#### Adherence to the MedDiet during the Confinement

Adherence to the MedDiet was assessed on the continuous scale (range: 0–14) and on the categorical scale by classifying participants into low, medium and high adherence levels (<5, 6 to 8 and >9 points, respectively) to the MedDiet at the two time points: before and during the COVID-19 Spanish confinement. A binary variable to assess the change in adherence to the MedDiet was built to distinguish between those who kept adherence to the MedDiet alike (reference category, set to zero) and those who changed their adherence towards a greater adherence (set to one). Logistic regression models were used to explore variables associated with the change in adherence (change versus non-change, as reference) to the MedDiet. Odds ratios (ORs) and corresponding 95% Confidence Intervals (CIs) were estimated in univariate regression models (Model 1), as well as in multivariate-adjusted models (Model 2: age, sex and center-adjusted; Model 3: Model 2 and physical activity, educational level and residence; to remove their influence on the dietary-MedDiet associations). To evaluate the effect of MedDiet-related variables on the change in adherence, mutual adjustment for all other MedDiet food items was performed in an additional model. The association between lifestyle and dietary variables with adherence to the MedDiet score (high versus medium-low, as reference) during confinement was also explored in logistic regression models. Model fit assumptions (Hosmer-Lemeshow test *p*-value > 0.05) were met. 

We tested potential effect modification by duration of confinement in days or weeks on the associations (between every variable and the change in adherence to the MedDiet) by adding interaction terms (combining variables with days/weeks) in the regression models. Linearity of the association between days and change in adherence to the MedDiet was evaluated using restricted cubic splines with three knots (percentiles 10, 50, and 90). The association with adherence to MedDiet over time was also explored through splines. Models with and without the interaction terms or splines were compared by means of the likelihood ratio test. Effect modification by sex and center was likewise tested. 

Several sensitivity analyses were performed to check the robustness of the results: (i) by removing survey respondents of the first week to minimize the effect of short-term changes in dietary habits on the associations; (ii) by evaluating the relative change in adherence to the MedDiet due to the COVID-19 confinement as “current-previous/current” adherence to the MedDiet. Duration of confinement and other variables associated with this relative change in adherence to the MedDiet (high versus low, as reference) were explored through logistic regression models considering the same multivariate adjusted models; (iii) by considering a modified scoring for assessing adherence to the MedDiet before the confinement, by incorporating information on intake of fried food and snacking. 

The threshold for statistical significance in two-sided tests was set at *p*-value = 0.05. Data were analyzed with R-project (version 3.4.1).

## 3. Results

### 3.1. Adherence to the Mediterranean Diet during the COVID-19 Confinement

A total of 7514 Spanish adults completed the questionnaire. The main socio-demographic characteristics of questionnaire respondents by levels of adherence (low, medium and high) to the MedDiet during the COVID-19 Spanish confinement are described in Table 1. 

**Figure 1:**
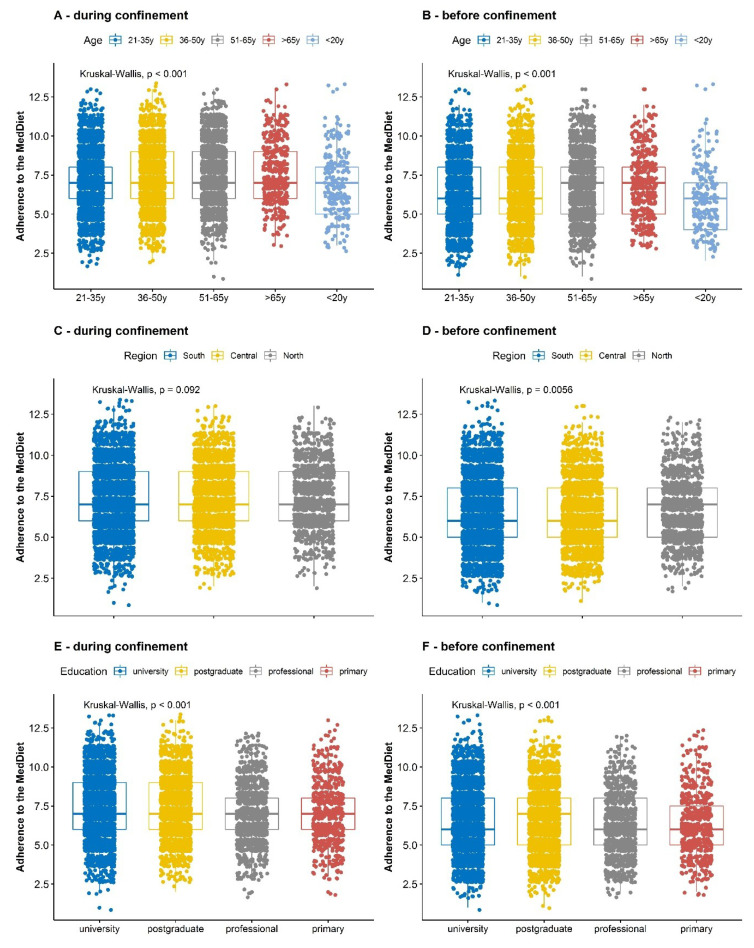
Adherence to the MedDiet before (**B**, **D** and **F**) and during (**A**, **C** and **E**) the Spanish COVID-19 confinement by subgroups of age (**A** and **B**), region (**C** and **D**) and educational level (**E** and **F**). In the box plots, the boundary of the box closest to zero indicates the 25th percentile, a colour line within the box marks the median, and the boundary of the box farthest from zero indicates the 75th percentile. Points above and below the box indicate the 10th and 90th percentiles; those above and below the whiskers indicate outliers. Numbers of included participants per group are shown in Table 1 and Table 2. Differences in mean adherence by groups were evaluated by means of the Kruskal-Wallis test.

About 71% of the participants were females, 53.6% were from the south of Spain and the majority attained a graduate (46.4%) or postgraduate education (31.5%). There were few participants in the youngest (3%) and oldest age groups (6%), whereas 92% of them were aged 31–65 years. Participants with higher adherence to the MedDiet (Table 1 and Figure 1) were more likely females, those living in family homes, in the mid-age groups (51–65 y) and with higher educational level i.e., university or postgraduate students. No differences by regions across MedDiet adherence levels were noted. Overall, mean adherence to MedDiet during the confinement was 7.34 (median = 7.0, range = 1–13). 

Dietary and lifestyle adaptations by level of adherence to the MedDiet during the Spanish confinement are shown in Table 2. Overall, most participants decreased their intake of alcohol (57.3%) and their physical activity level (59.6%) during the confinement. Furthermore, the majority cooked in a similar way than before the confinement and used the griddle as the main technique for cooking (44.4%). Eating small amounts of food between meals (snaking), the intake of fried foods and fast-food were also similar than before the COVID-19 confinement, and 63.7% of participant declared not to have been eating more during the confinement. Around 73% of participants kept their intake of fried foods as before the COVID-19 confinement, which meant that nearly 39% of them continued consuming fried foods 1–3 days a week and around 37% less than 1 time per week. The majority of the participants (68.4%) used olive oil for frying. Interestingly, among total participants, around 27% had difficulties finding some types of foods, especially meat (23.83%), vegetables (13.86%) and fish (12.11%) during the COVID-19 confinement. A higher adherence to MedDiet was observed among those who used not to eat out of home and among those who reduced or kept their intake of fried food, fast food and snacking frequency, as well as those who kept being active and maintained the same weight. MedDiet MEDAS-derived food items were consistently related to high MedDiet adherence levels during the confinement (Supplementary Table S2). These variables were also significantly associated with adherence to the MedDiet during confinement in regression models comparing high versus medium-low adherence (Supplementary Table S3).

### 3.2. Adherence to the Mediterranean Diet before the COVID-19 Confinement

Differences between MedDiet-related dietary behaviours during the COVID-19 Spanish confinement were found (Table 3). Since the beginning of confinement, participants with higher adherence to the MedDiet decreased the intake of sweet/carbonated beverages, red meat and pastries by 16–18%. By contrast, the intake of fruits and vegetables increased by around 12% in the high MedDiet adherence group. Mean adherence to the MedDiet before the confinement was 6.53 (median = 6.0, range= 1–13). Thus, adherence to the MedDiet increased from the pre to post-confinement period, with this increase being statistically significant (*p*-value = <0.001). The relation between MedDiet MEDAS-derived food items MedDiet adherence levels before the confinement was also consistent (Supplementary Table S4).

Age, educational level and region were related to significant differences in adherence before and during confinement (Figure 1B,D,F). Concretely, participants with ages between 21 to 50 years old showed a significantly lower adherence (*p* < 0.001) to the MedDiet compared to participants of age > 51 years. Questionnaire respondents with higher educational levels (post-graduate diploma or doctorate) also showed a higher adherence to the MedDiet (*p* < 0.001) before the confinement. Participants from the north of Spain seemed to have a higher adherence to the MedDiet compared to the other geographical regions.

### 3.3. Changes in Lifestyle and Dietary Behaviours during the COVID-19 Confinement

There were 3392 survey respondents who changed their adherence to the MeDiet. In all of them, this change was related to an increase in adherence to MedDiet. Socio-demographic factors associated with the change in the adherence to the MedDiet are presented in Supplementary Table S5. Multivariate adjusted models revealed that participants who lived in the north of Spain (OR: 0.67), with children in care and with ages ranging from 51 to higher than 65 years old (OR < 0.90) presented a lower odd of change to adhere to the MedDiet during the Spanish confinement. On the contrary postgraduate respondents (OR: 1.13, 95% IC: 1.02–1.26) and those who lived alone (OR: 1.36, 95% IC: 1.17–1.58) had a greater likelihood of increasing their adherence to the MedDiet due to the confinement.

By dietary and lifestyle habits (Table 4), several food choices were associated with the change in adherence to the MedDiet during the confinement. Multivariate-adjusted models showed that those participants who reported a lower intake of fried foods, alcohol, fast-food, and snacks during the COVID-19 Spanish confinement had a statistically significant higher likelihood of turning into a higher adherence to the MedDiet compared to those who kept their usual intake as before the confinement (OR: 4.71; 2.15; 3.12; 3.53, respectively). Compared to respondents who used not have any daily meal out of home, the OR associated with change in adherence to MedDiet increased significantly in respondents who had one or more daily meals out of home. Also, compared to those who kept being active, inactive respondents showed a significantly lower odds (OR: 0.78; 95% CI: 0.62–0.97) of changing the adherence to the MedDiet. Interestingly, these variables were associated with a lower adherence to the MedDiet during confinement (Supplementary Table S3). Among those who changed their adherence to MedDiet, there were 48.2% respondents who did not gain weight since confinement, compared to 13% who gained weight (OR: 1.01, *p*-value > 0.05) and 38.6% who were unsure about weight changes (OR: 0.89; *p*-value < 0.05). No association was observed between the change in MedDiet adherence and the type of cooking or eating more behaviour (data not shown), or by week of confinement. 

Table 5 displays multivariate-adjusted ORs associated with the change in adherence to MedDiet in relation to the MEDAS-derived foods, considering mutual adjustment by each other food item. Results showed that higher intake of MedDiet typical foods such as vegetables (OR: 8.08, 95% CI: 6.84–9.50), fish (OR: 6.17, 95% CI: 4.98–7.69), olive oil (OR: 5.21, 95% CI: 4.24–6.39), and legumes (OR: 4.71, 95% CI: 4.04–5.46) during the COVID-19 confinement, were associated with a higher likelihood of changing the adherence to the MedDiet. Lower intake of non-typical MedDiet foods such as red meat, sweetened beverages or non-homemade pastries were also significantly associated with the change in MedDiet adherence. The association could not be evaluated for other food items, for which information on changes in intake was not collected (sofrito, wine, fats, nuts and white meat preference). There was no evidence for effect modification by duration of confinement on the associations (data not shown). The geographical region was also not found to modify the associations (data not shown). However, a statistically significant interaction by gender was observed between change in adherence to the MedDiet and the intakes of fruits, vegetables and olive oil (*p*-value for interaction = 0.016, 0.001 and 0.006, respectively). Indeed, men showed a stronger association between the change in adherence to MedDiet and these variables than women (e.g., OR for the association between increase of olive oil intake and MedDiet adherence change = 8.00 in men and 3.71 in women) (Supplemental Table S6). 

Given the linearity of the association between MedDiet adherence change and duration of confinement (Supplementary Figure S1A), it was estimated that the odds of change in the adherence increased by 8% per 5 days increase of confinement (95% CI: 1.01–1.17). No significant difference was seen in either adherence to the MedDiet score or change in the adherence to the MedDiet by weeks of confinement (Supplementary Figure S1B). Yet a non-linear association between adherence to the MedDiet during confinement and days of confinement was apparent (Supplementary Figure S1C), supporting that the adherence to this dietary pattern during confinement increased up to 15 days of confinement and tended to decrease thereafter. This trend might lack consistency as there were fewer respondents in the last week.

Results on the association between change in adherence to the MedDiet and dietary/lifestyle variables remained unchanged in sensitivity analyses (Supplementary Table S7).

## 4. Discussion

The current study is the first reporting that dietary changes towards a healthier diet have taken place during the COVID-19 confinement. Our findings illustrate how the Spanish adult population have adopted healthier dietary behaviours during the COVID-19 confinement by means of a closer approach to the MedDiet-style eating patterns. The adherence to the MedDiet, measured by the 14-points MEDAS, increased by 0.8 points during the three first weeks of the Spanish confinement period, but seemed to decrease slightly at a later stage. 

The MedDiet, considered as a reference of a healthy eating approach, is dominated by the intake of olive oil and by high consumption of vegetables and fruits. It has traditionally been highlighted to contribute to the good health of the Mediterranean people, and nowadays there is consistent evidence supporting its association with lower all-cause mortality and reduced risk of CVD [19,20]. Furthermore, the MedDiet-related foods have been recently recommended to be included in our diet during the COVID-19 confinement due their capacity to strengthen the immune system [16].

Overall, data from this study showed medium adherence to the MedDiet (mean of MEDAS score 6.53 ± 2) in the studied population before the COVID-19 outbreak that significantly increased (mean of MEDAS score 7.34 ± 1.93) during the confinement. It could be argued that Spain, a Mediterranean country, should have a higher adherence to the MedDiet. However, data from this study are consistent with those recently published by Santi-Cano et al. [21] who reported a MedDiet average score of 6.2 ± 1.8 points in young adults, i.e., 275 university students from the South of Spain with mean age of 22.2 ± 6.3 years old. In fact, an abandonment by the MedDiet by the Spanish adults was earlier documented by León-Muñoz et al. in 2012 [22]. Our results also agreed with those found by the same author, i.e., mean of MEDAS score 6.34 ± 0.03 in a cross-sectional study that included 11,742 adults, representative of the Spanish population [22]. 

Adherence to the MedDiet before and during the COVID-19 confinement was also significantly influenced by regions, place of residence and educational level. In this regard, the adherence to the MedDiet was significantly higher in northern Spanish countries than in southern or central ones before the COVID-19 confinement. The DIMERICA study observed lower adherence to the MedDiet of participants from Southeastern Spain in a descriptive cross-sectional study including 1732 subjects from different Spanish regions [23]. Nevertheless, the different areas that they included according to geographical region, are not comparable with ours. Those significant differences, however, were not observed during the COVID-19 confinement.

As expected, higher adherence to the MedDiet was also found in participants lived in the family home compared to those who lived alone at the time of being surveyed, respectively. Living in the family home has been associated with a higher quality of diet [24]. Additionally, higher educational level has been commonly associated with higher socioeconomic status which, at the same time, has been related to a better diet quality [25]. In agreement with our results, Cavaliere et al. [26] found a positive link between education (and income) and the adherence to the MedDiet, which has been highlighted to be more expensive than the traditional Western diet [27].

It was not surprising that participants from our study aged from 51 years and older presented higher adherence to the MedDiet before the COVID-19 pandemic started, compared to the other age groups. In agreement with our results, higher adherence to the MedDiet in Spanish adults from 45 years and older was previously found, compared to the youngest ones [22]. The adherence to the MedDiet in the group of age from 51 years and older was slightly but significantly increased during the confinement in our study. Interestingly, the highest change was found in the youngest survey respondents who also significantly increased the adherence to the MedDiet during the confinement. The mean MEDAS score raised by 0.92 points up to 6.86 ± 2.14. When we looked at the MedDiet related-foods contributing most to the change in the MedDiet adherence during the COVID-19 confinement, a lower intake of pastries, red meat and sweetened/carbonated beverages followed by a higher intake of vegetables, fruits and olive oil were the most important contributors to this association. The lower consumption of meat could be related with the lack of stock in the Spanish supermarkets and grocery stores after the state of alarm was declared. In fact, nearly 28% of participants who had any difficulty finding any food in their usual supermarket highlighted meat as the main one. COVID-19 related changes in consumer behaviours have been noticed in Spain. As an example, online grocery sales have skyrocketed by 25% during the last weeks after the state of alarm was declared, according to data from the European Foundation for Innovation (INTEC). In the same report, the demand for confectionery products and butter also increased in more than 50% [28]. This is in agreement with the latest data from the Google Trends tool which shows how the Spanish population has considerably increased their search about the term “homemade cake” coinciding with the first week of confinement in March (when the ‘State of Alarm’ began) and compared to the last five years (Figure S2a). However, it does not explain that most people in our study decreased the intake of pastries (both, commercial and homemade pastries) during the confinement. It could be assumed that during the confinement people could have more time for cooking. In spite of according to Google Trends the term “traditional recipes” also increased exponentially (Figure S2b), data about the influence of cooking in the increase of adherence to the MedDiet during the confinement were somehow contradictory. Particularly, participants who increased or decreased cooking during the confinement, were more likely to change their adherence to the MedDiet (OR: 1.83; 95% CI: 1.67–2.01 and OR: 2.01; 95% CI: 1.57–2.58) compared to those who kept cooking as before the confinement starts. For those who cooked less during that period, the explanation of a higher association with the adherence to the MedDiet could reside in the choice of fresh foods typical from the MedDiet which can be eaten raw such as vegetables and preserved and cooked legumes. For those participants who cooked more during the confinement than they used to do, it can be assumed that they were adopting healthier cooking habits than they had before the confinement.

The intake of wine was not associated with a higher adherence to the MedDiet before or during the confinement. Wine is a well-known component of the Mediterranean diet and its moderate intake has been traditionally associated with CVD risk reduction. However, the latest evidence suggests that its association is not clear enough yet [29]. Data from the DiSA-UMH prospective cohort which includes 1204 university students aged 17 to 35 years concluded that low to moderate wine/beer consumers had higher adherence to the MedDiet compared to non-drinkers or drinkers of all alcoholic beverages further than beer and wine [30]. On the contrary, the SUN cohort failed to find a relationship between wine or other alcoholic beverages and higher adherence to the MedDiet in 10526 Spanish university graduates with ages from 18–95 years [31]. Data from our study showed that lower alcohol intake during the confinement, including not only wine but also beer and high-grade drinks, was associated with a higher adherence to the MedDiet. However, the lack of quantitative data about alcohol intake before and during the COVID-19 confinement, prevent us from making direct comparisons with them.

A higher likelihood of having an increased adherence to the MedDiet was found among those participants who used to have 2 or more meals out of home daily compared to participants who had any or just one of the main meals (breakfast, lunch or dinner) out of home daily before the confinement. Eating out of home increased rapidly in the Mediterranean countries due to changes in the socio-economic systems [32] and even today is a common practice among the Spanish population, according to the last report from the Spanish Ministry of Agriculture, Fisheries and Food [13]. A systematic review aimed to study the association with dietary intake eating out of home, concluded that eating out of home contributes to a higher intake of fats, particularly saturated fats in the daily diet especially in adults [33]. Llanaj et al. [34] reported a higher intake of foods that do not fit the traditional MedDiet such as sweets, soft drinks and meat products and a low consumption of fruits and vegetables among undergraduate students who used to eat out of home. 

Finally, a moderate intake of fried foods was maintained by the majority of participants during the confinement. We observed that participants who reported lower intake of fried foods and those with lower frequency of intake (<1 time/week or never) during the COVID-19 confinement were more prone to increase their adherence to the MedDiet. In this regard, higher frequency of MedDiet-related foods such as vegetables or fruits were found in participants who consumed fried foods 0–2 times/week compared to those with higher intakes in the SUN cohort in which 13,679 participants were included [35]. As expected, the main oil employed for frying was olive which agrees with three of the main studies based on the Spanish population i.e., the SUN cohort study [36], Pizarra study [37] and EPIC study [38]. It should be mention that the SUN study reported a higher frequency of intake of fried foods compared to our results. However, the mean age of the SUN project population was lower than the COVIDiet population is and the survey is from about 10 years ago [36]. 

Strengths of our study are many. To the best of our knowledge, this study is the first in examining dietary behaviours and changes in the adherence to the MedDiet under a new situation such as the lockdown caused by the COVID-19 pandemic. The online questionnaire allowed for rapid and cost-efficient assembly of self-reported information about dietary behaviours. It was useful to achieve a relatively large number of participants which had been impossible to obtain employing face-to-face interviews due to the COVID-19 confinement. Additionally, the COVIDiet questionnaire allowed us to collect a high amount of information referred to eating and healthy lifestyle behaviours, including the MedDiet dietary pattern, before and during the confinement.

Some limitations should also be acknowledged. Among them, the oversampling of a particular network due to the non-random snowball sampling method could be highlighted. For example, respondents were predominantly from Andalusia and 70.6% of participants were women. The same selection bias could be found with the level of education and income. However, with this respect, Ekman et al. [39] affirmed that the bias associated with collecting information using online questionnaires was not greater than that caused by paper questionnaires. The low representation of people with primary studies in the COVIDiet study (8.2%) in relation with the whole population could be due to the fact that this population is less likely to use smart technologies could have been an obstacle to participate in the survey. Nevertheless, it should be pointed out that, despite the current research could not be representative of all the Spanish adult population, coverage of all age groups, Spanish territories and educational levels has been achieved. Since non-personal data were included in the questionnaire, no questions regarding job titles/categorizations were included. For that reason, it cannot be excluded that some of the survey respondents (e.g., pharmacists, health care providers, people who work in supermarkets or grocery stores, drivers, shippers, etc.) were not completely locked down. However, it should be mentioned that those persons had to eat at home due to the impossibility to have lunch outside because all restaurants were closed. Since the participants were untrained, the perception that they could had about the food portions could underestimate/overestimate their real intake. Additionally, since we performed a web-based study, in which a questionnaire was self-administered and online, participants did not have the opportunity to ask about any doubts that could be presented. However, the MEDAS is a validated and widely used instrument for rapid estimation to the MedDiet adherence [17] which allows further comparisons to other research studies. The participation of non-healthy persons who require to follow a special diet cannot be discarded; this information was, however, not collected. Finally, while we conducted several statistical tests, all were hypothesis-driven as we analyzed a predetermined number of variables assumed to be associated with dietary habits in the population. Multiple testing issues in our study are therefore unlikely. 

## 5. Conclusions

This is the first study focused on evaluating changes in food consumption habits in a Spanish adult population during the COVID-19 outbreak confinement, based on a web-based survey targeted to the adult general population. From this study, we can conclude that the studied Spanish adult population is still far away from having good healthy dietary habits considering the MedDiet as reference of healthy eating. However, an improvement of their dietary behaviours during the COVID-19 confinement has been observed. Health-related food choices included higher intake of fruits, vegetables or legumes and lower intake of red meat, alcohol, fried foods or pastries compared to their usual habits. In spite of the observed change towards a healthier dietary pattern, permanent dietary habits are difficult to maintain. This improvement, if sustained in the long-term, could have a positive impact on the prevention of chronic diseases and COVID-19-related complications, and should be therefore promoted.

## Figures and Tables

**Table 1:** Baseline characteristics of questionnaire respondents by level of adherence to the MedDiet during the COVID-19 Spanish confinement.

		All (N = 7514)		Low (N = 1275)		Medium (N = 4129)		High (N = 2110)		*p*-Value ^3^
		N	%	N	%	N	%	N	%	
**Gender ^1^**										<0.001
	Men	2204	29.3	444	34.8	1195	28.9	565	26.8	
	Women	5305	70.6	830	65.1	2931	71.0	1544	73.2	
**Place of Residence**										0.01
	Family home	6150	81.8	1030	80.8	3355	81.3	1765	83.6	
	Shared flat	535	7.1	109	8.6	302	7.3	124	5.9	
Student residence		31	0.4	5	0.4	23	0.6	3	0.1	
	Alone	798	10.6	131	10.3	449	10.9	218	10.3	
**Region by Areas ^2^**										0.52
	Central	2266	30.2	386	30.3	1242	30.1	638	30.2	
	North	1223	16.3	193	15.1	665	16.1	365	17.3	
	South	4025	53.6	696	54.6	2222	53.8	1107	52.5	
**Children in Care**										0.01
	No	4503	59.9	811	63.6	2446	59.2	1246	59.1	
	Yes	3011	40.1	464	36.4	1683	40.8	864	40.9	
**Educational Level**										<0.001
	University	3487	46.4	559	43.8	1923	46.6	1005	47.6	
	Postgraduate	2370	31.5	367	28.8	1273	30.8	730	34.6	
	Professional	1042	13.9	221	17.3	582	14.1	239	11.3	
	Primary	615	8.2	128	10.0	351	8.5	136	6.5	
**Age (Years)**										<0.001
	21–35	2558	34.0	551	43.2	1368	33.1	639	30.3	
	36–50	2371	31.6	377	29.6	1316	31.9	678	32.1	
	51–65	1928	25.7	232	18.2	1097	26.6	599	28.4	
	>65	428	5.7	51	4.0	234	5.7	143	6.8	
	<20	229	3.0	64	5.0	114	2.8	51	2.4	
**Week ^4^**	1	1510	20.1	244	19.1	855	20.7	411	19.5	0.68
	2	5167	68.8	886	69.5	2815	68.2	1466	69.5	
	3	837	11.1	145	11.4	459	11.1	233	11.0	
		**All (N = 7514)**		**Low (N = 1275)**		**Medium (N = 4129)**		**High (N = 2110)**		***p*-Value**
		**Mean**	**SD**	**Mean**	**SD**	**Mean**	**SD**	**Mean**	**SD**	
**Days ^4^**		10.1	3.3	10.1	3.4	10.1	3.3	10.2	3.3	0.171
**Weight (kg)**		68.8	16.3	70.8	15.6	68.9	14.4	67.6	19.8	<0.001
**Adherence to MedDiet ^5^**										
Before confinement		6.5	2.0	4.2	0.9	6.2	1.2	8.7	1.6	<0.001
During confinement		7.3	1.9	4.5	0.7	7.0	0.8	9.8	0.9	<0.001

^1^ Numbers do not add up because there were five respondents who reported another gender (data not shown), ^2^ Spanish Autonomous regions were grouped into areas: Central Spain (Castilla y León 4.3%, Madrid 12.5%, Aragón 1.2%, Castilla La Mancha 2%, Extremadura 1.7%, Islas Baleares 2%; Comunidad Valenciana 4.5%. Murcia 2.1%). Northern Spain (Cantabria 0.4%. Asturias 0.7%. Galicia 2.3%, País Vasco 6.8%, Cataluña 4.9%, Navarra 1%, La Rioja 0.2%), Southern Spain (Andalucía 50.4%, Ceuta 0.1%, Melilla 1.2%, Islas Canarias 2%), ^3^ Differences between the three MedDiet adherence groups were evaluated by the Chi-squared test, ^4^ Weeks or days of confinement, ^5^ Based on the Mediterranean Diet Assessment Score (MEDAS).

**Table 2:** Dietary and lifestyle adaptations by level of adherence to the MedDiet during the COVID-19 Spanish confinement.

	All (N = 7514)		Low (N = 1275)		Medium (N = 4129)		High (N = 2110)		*p*-Value ^1^
	N	%	N	%	N	%	N	%	
**Meals Out-of-Home ^2^**									<0.001
0	2975	39.6	387	30.4	1600	38.8	988	46.8	
1	2207	29.4	392	30.7	1245	30.2	570	27.0	
2	758	10.1	176	13.8	425	10.3	157	7.4	
3	1574	20.9	320	25.1	859	20.8	395	18.7	
**Alcohol Intake**									<0.001
As before	2429	32.3	384	30.1	1265	30.6	780	37.0	
Lower	4302	57.3	757	59.4	2390	57.9	1155	54.7	
Higher	783	10.4	134	10.5	474	11.5	175	8.3	
**Type of Cooking**									<0.001
Stew	2208	29.4	302	23.7	1258	30.5	648	30.7	
Fried	310	4.1	122	9.6	148	3.6	40	1.9	
Oven	1398	18.6	249	19.5	750	18.2	399	18.9	
Microwave	261	3.5	73	5.7	125	3.0	63	3.0	
Griddle	3337	44.4	529	41.5	1848	44.8	960	45.5	
**Frequency of Cooking**									<0.001
As before	3809	50.7	594	46.6	2055	49.8	1160	55.0	
Lower	273	3.6	73	5.7	147	3.6	53	2.5	
Higher	3432	45.7	608	47.7	1927	46.7	897	42.5	
**Fried Foods Intake**									<0.001
As before	5517	73.4	887	69.6	2995	72.5	1635	77.5	
Lower	1525	20.3	217	17.0	885	21.4	423	20.0	
Higher	472	6.3	171	13.4	249	6.0	52	2.5	
**Fried Foods Frequency Per Week**									<0.001
1–3	2894	38.5	647	50.7	1656	40.1	591	28.0	
4–6	309	4.1	105	8.2	157	3.8	47	2.2	
>7	23	0.3	11	0.9	7	0.2	5	0.2	
<1	2755	36.7	370	29.0	1521	36.8	864	40.9	
Never	1533	20.4	142	11.1	788	19.1	603	28.6	
**Oil Used for Frying**									<0.001
Olive oil	5139	68.4	891	69.9	2848	69.0	1400	66.4	
Sunflower	930	12.4	235	18.4	546	13.2	149	7.1	
None	1383	18.4	129	10.1	709	17.2	545	25.8	
Other	62	0.8	20	1.6	26	0.6	16	0.8	
**Snacking Frequency**									<0.001
As before	3511	46.7	483	37.9	1923	46.6	1105	52.4	
Lower	1176	15.7	150	11.8	658	15.9	368	17.4	
Higher	2827	37.6	642	50.4	1548	37.5	637	30.2	
**Fast Food Frequency**									<0.001
As before	4507	60.0	627	49.2	2466	59.7	1414	67.0	
Lower	2623	34.9	498	39.1	1471	35.6	654	31.0	
Higher	384	5.1	150	11.8	192	4.7	42	2.0	
**Eating More**									<0.001
No	4783	63.7	637	50.0	2600	63.0	1546	73.3	
Yes	2731	36.3	638	50.0	1529	37.0	564	26.7	
**Physical Activity**									<0.001
As before	1403	18.7	191	15.0	766	18.6	446	21.1	
Lower	4475	59.6	770	60.4	2458	59.5	1247	59.1	
Higher	1196	15.9	187	14.7	652	15.8	357	16.9	
Never	440	5.9	127	10.0	253	6.1	60	2.8	
**Weight Gain**									<0.001
No	3556	47.3	464	36.4	1918	46.5	1174	55.6	
Unknown	2993	39.8	593	46.5	1663	40.3	737	34.9	
Yes	965	12.8	218	17.1	548	13.3	199	9.4	

^1^ Differences between the three MedDiet adherence groups were evaluated by the Chi-squared test. ^2^ Number of daily meals out of home before the confinement.

**Table 3:** Comparisons between dietary behaviours relative to the MedDiet pattern by level of adherence to the MedDiet before and during the COVID-19 Spanish confinement.

MEDAS Food Groups	Low Before (N = 2447)		Low Confinement (N = 1275)		High Before (N = 1294)		High Confinement (N = 2135)	
	N	%	N	%	N	%	N	%
**Olive Oil (Tablespoons/d)**								
As before	1921	78.5	1075	84.3	1222	94.4	1850	87.7
Lower	225	9.2	72	5.7	39	3	117	5.5
Higher	301	12.3	128	10.0	33	2.6	143	6.8
**Vegetables (Servings/d)**								
As before	1462	59.7	878	68.9	1157	89.4	1583	75
Lower	416	17	248	19.5	69	5.3	113	5.4
Higher	569	23.3	149	11.7	68	5.3	414	19.6
**Fruits (Servings/d)**								
As before	1395	57	830	65.1	1092	84.4	1523	72.2
Lower	432	17.7	230	18.0	114	8.8	197	9.3
Higher	620	25.3	215	16.9	88	6.8	390	18.5
**Red Meat (Servings/d)**								
As before	1278	52.2	919	72.1	1160	89.6	1505	71.3
Lower	915	37.4	167	13.1	80	6.2	528	25.0
Higher	254	10.4	189	14.8	54	4.2	77	3.7
**Sweet Beverages (Servings/d)**								
As before	1201	49.1	834	65.4	1203	93	1592	75.5
Lower	959	39.2	228	17.9	53	4.1	459	21.8
Higher	287	11.7	213	16.7	38	2.9	59	2.8
**Legumes (Servings/w)**								
As before	1706	69.7	975	76.5	1165	90	1688	80.0
Lower	280	11.4	140	11.0	35	2.7	95	4.5
Higher	461	18.8	160	12.5	94	7.3	327	15.5
**Fish (Servings/w)**								
As before	1546	63.2	907	71.1	1086	83.9	1575	74.6
Lower	639	26.1	278	21.8	172	13.3	376	17.8
Higher	262	10.7	90	7.1	36	2.8	159	7.5
**Non-Homemade Pastries (Units/w)**								
As before	1031	42.1	661	51.8	1084	83.8	1450	68.7
Lower	767	31.3	155	12.2	70	5.4	450	21.3
Higher	649	26.5	459	36.0	140	10.8	210	10.0

Differences between the two MedDiet adherence groups (low and high) were evaluated by the Chi-squared test. Differences between MedDiet adherence levels, before and during the confinement, were all statistically significant at *p* < 0.001 level.

**Table 4:** Associations between eating and lifestyle habits and the change in the adherence to the MedDiet during the COVID-19 Spanish confinement.

	Non-Change N = 4122		Change in MD Adherence		*p*-Value ^1^	Crude Model ^2^		Adjusted Model ^3^	
			N = 3392						
	N	%	N	%		OR	95% CI	OR	95% CI
**Meals out-of-home ^2^**					<0.001				
0	1771	43.0	1204	35.5		Ref.		Ref.	
1	1198	29.1	1009	29.7		**1.24**	**[1.11;1.38]**	**1.20**	**[1.07;1.35]**
2	340	8.2	418	12.3		**1.81**	**[1.54;2.12]**	**1.72**	**[1.45;2.02]**
3	813	19.7	761	22.4		**1.38**	**[1.22;1.56]**	**1.32**	**[1.16;1.50]**
**Alcohol intake**					<0.001				
As before	1612	39.1	817	24.1		Ref.		Ref.	
Lower	2062	50.0	2240	66.0		**2.14**	**[1.93;2.38]**	**2.15**	**[1.93;2.38]**
Higher	448	10.9	335	9.9		**1.48**	**[1.25;1.74]**	**1.42**	**[1.20;1.68]**
**Difficult finding foods**					<0.001				
No	3076	74.6	2392	70.5		Ref.		Ref.	
Yes	1046	25.4	1000	29.5		**1.23**	**[1.11;1.36]**	**1.19**	**[1.07;1.32]**
**Frequency of cooking**					<0.001				
As before	2372	57.5	1437	42.4		Ref.		Ref.	
Lower	123	3.0	150	4.4		**2.01**	**[1.57;2.58]**	**1.98**	**[1.54;2.55]**
Higher	1627	39.5	1805	53.2		**1.83**	**[1.67;2.01]**	**1.78**	**[1.61;1.96]**
**Fried foods intake**					<0.001				
As before	3452	83.7	2065	60.9		Ref.		Ref.	
Lower	397	9.6	1128	33.3		**4.75**	**[4.19;5.39]**	**4.71**	**[4.14;5.35]**
Higher	273	6.6	199	5.87		**1.22**	**[1.01;1.47]**	1.18	[0.97;1.43]
**Fried foods frequency per week**					0.001				
1–3	1665	40.4	1229	36.2		Ref.		Ref.	
4–6	180	4.4	129	3.8		0.97	[0.76;1.23]	0.97	[0.76;1.23]
>7	14	0.3	9	0.3		0.88	[0.36;2.02]	0.85	[0.36;1.99]
<1	1475	35.8	1280	37.7		**1.18**	**[1.06;1.31]**	**1.15**	**[1.04;1.28]**
Never	788	19.1	745	22.0		**1.28**	**[1.13;1.45]**	**1.21**	**[1.06;1.37]**
**Oil used for frying**					0.123				
Olive oil	2865	69.5	2274	67.0		Ref.		Ref.	
Sunflower	497	12.1	433	12.8		1.10	[0.95;1.26]	1.12	[0.97;1.29]
None *	725	17.6	658	19.4		**1.14**	**[1.02;1.29]**	1.08	[0.95;1.22]
Other	35	0.8	27	0.8		0.97	[0.58;1.61]	0.97	[0.58;1.61]
**Snacking frequency**					<0.001				
As before	2179	52.9	1332	39.3		Ref.		Ref.	
Lower	373	9.0	803	23.7		**3.52**	**[3.06;4.05]**	**3.53**	**[3.06;4.07]**
Higher	1570	38.1	1257	37.1		**1.31**	**[1.18;1.45]**	**1.29**	**[1.16;1.43]**
**Fast food frequency**					<0.001				
As before	2918	70.8	1589	46.8		Ref.		Ref.	
Lower	973	23.6	1650	48.6		**3.11**	**[2.82;3.44]**	**3.12**	**[2.82;3.46]**
Higher	231	5.6	153	4.5		1.22	[0.98;1.50]	1.20	[0.96;1.49]
**Physical activity**					0.022				
As before	768	18.6	635	18.7		Ref.		NA	
Lower	2460	59.7	2015	59.4		0.99	[0.88;1.12]		
Never	268	6.5	172	5.1		**0.78**	**[0.62;0.97]**		
**Weight gain**					0.135				
No	1920	46.6	1636	48.2		Ref.		Ref.	
Unknown	1684	40.9	1309	38.6		0.91	[0.83;1.01]	**0.89**	**[0.80;0.98]**
Yes	518	12.6	447	13.2		1.01	[0.88;1.17]	1.01	[0.87;1.17]
**Week**					0.135				
1	825	20.0	685	20.2		Ref.		Ref.	
2	2864	69.5	2303	67.9		0.97	[0.86;1.09]	0.97	[0.86;1.09]
3	433	10.5	404	11.9		1.12	[0.95;1.33]	1.13	[0.94;1.36]

^1^ Differences between the groups were evaluated by the Chi-squared test. Statistical significance, ^2^ Crude model: unadjusted for any variable, ^3^ adjusted model: adjusted for gender (men. women. other). Age groups (<20. 20–35 y. 25–50 y. 50–65 y. >65 y). Regions (Central. Northern and Southern). Educational level (primary. professional. university. postgraduate). Residence (family home. shared flat. residence. alone). Physical activity (similar. higher. lower. never), * this category included non-consumers of fried foods, but also includes users of other types of oil or fats for frying and “pan frying”. Statistically significant ORs are highlighted in bold.

**Table 5:** Influence of the MEDAS-derived foods intake on the change in the adherence to the MedDiet during the COVID-19 Spanish confinement.

	Non-Change N = 4122		Change in MD Adherence N = 3392		*p*-Value ^1^	Adjusted Model ^2^	
	N	%	N	%		OR	95% CI
**Olive Oil for Cooking**					0.187		
No	66	1.60	69	2.0		Ref.	
Yes	4056	98.4	3323	98.0		0.70	[0.49;1.01]
**Olive Oil (Tablespoons/d)**					0.573		
>4	1162	28.2	987	29.1		Ref.	
0–1.9	732	17.8	612	18.0		0.93	[0.81;1.08]
2–3.9	2228	54.1	1793	52.9		0.91	[0.82;1.02]
**Olive Oil Confinement**					<0.001		
As before	3831	93.0	2633	77.6		Ref.	
Lower	150	3.6	319	9.4		**3.22**	**[2.61;3.96]**
Higher	141	3.4	440	13.0		**5.21**	**[4.24;6.39]**
**Vegetables (Servings/d)**					<0.001		
>2	1197	29.0	1362	40.2		Ref.	
0–0.9	790	19.2	486	14.3		**0.69**	**[0.59;0.81]**
1–1.9	2135	51.8	1544	45.5		**0.73**	**[0.66;0.82]**
**Vegetables Confinement**					<0.001		
As before	3461	84.0	2001	59.0		Ref.	
Lower	449	10.9	418	12.3		**1.92**	**[1.64;2.23]**
Higher	212	5.14	973	28.7		**8.08**	**[6.84;9.50]**
**Fruits (Servings/d)**					<0.001		
>3	867	21.0	984	29.0		Ref.	
0–0.9	893	21.7	577	17.0		**0.68**	**[0.58;0.80]**
1–2.9	2362	57.3	1831	54.0		**0.75**	**[0.66;0.84]**
**Fruits Confinement**					<0.001		
As before	3342	81.1	1842	54.3		Ref.	
Lower	471	11.4	531	15.7		**2.36**	**[2.03;2.72]**
Higher	309	7.5	1019	30.0		**6.49**	**[5.61;7.52]**
**Red Meat (Servings/d)**					<0.001		
>1	786	19.1	456	13.4		Ref.	
0–0.9	3336	80.9	2936	86.6		**1.32**	**[1.16;1.51]**
**Red Meat Confinement**					0.000		
As before	3766	91.4	1587	46.8		Ref.	
Lower	42	1.0	1590	46.9		**101.48**	**[74.66;140.66]**
Higher	314	7.6	215	6.3		**1.82**	**[1.50;2.23]**
**Fats (Servings/d)**					0.225		
>1	181	4.4	170	5.0		Ref.	
0–0.9	3941	95.6	3222	95.0		**0.73**	**[0.58;0.91]**
**Sweet Beverages (Servings/d)**					<0.001		
>1	468	11.4	254	7.5		Ref.	
0–0.9	3654	88.6	3138	92.5		1.35	[0.87;1.33]
**Sweet Beverages Confinement**					0.000		
As before	3745	91. 0	1600	47.2		Ref.	
Lower	30	0.7	1578	46.5		**145.47**	**[102.21;214.52]**
Higher	347	8.4	214	6.3		**1.90**	**[1.52;2.34]**
**Legumes (Servings/w)**					<0.001		
>3	652	15.8	797	23.5		Ref.	
0–0.9	727	17.6	482	14.2		**0.61**	**[0.52;0.72]**
1–2.9	2743	66.5	2113	62.3		**0.69**	**[0.61;0.77]**
**Legumes Confinement**					<0.001		
As before	3625	87.9	2247	66.2		Ref.	
Lower	209	5.1	330	9.7		**2.97**	**[2.47;3.62]**
Higher	288	7.0	815	24.0		**4.71**	**[4.04;5.46]**
**Fish (Servings/w)**					0.003		
>3	636	15.4	605	17.8		Ref.	
0–0.9	1173	28.5	1001	29.5		1.06	[0.91;1.23]
1–2.9	2313	56.1	1786	52.7		0.91	[0.80;1.04]
**Fish Confinement**					<0.001		
As before	3403	82.6	2034	60.0		Ref.	
Lower	594	14.4	942	27.8		**2.88**	**[2.54;3.25]**
Higher	125	3.0	416	12.3		**6.17**	**[4.98;7.69]**
**Pastries (Unit/day)**					<0.001		
>2	1118	27.1	714	21.0		Ref.	
0–1.9	3004	72.9	2678	79.0		**1.28**	**[1.14;1.44]**
**Pastries Confinement**					0.000		
As before	3187	77.3	1540	45.4		Ref.	
Lower	76	1.8	1261	37.2		**36.59**	**[28.89;46.94]**
Higher	859	20.8	591	17.4		**1.68**	**[1.46;1.94]**

^1^ Differences between the groups were evaluated by the Chi-squared test. Statistical significance, ^2^ adjusted model: adjusted for gender (men. women. other). Age groups (<20. 20–35 y. 25–50 y. 50–65 y. >65 y). Regions (Central. Northern and Southern). Educational level (primary. professional. university. postgraduate). Residence (family home. shared flat. residence. alone). Physical activity (similar. higher. lower. never). In addition. ORs of the association between every food item and the MedDiet adherence change were mutually adjusted by each other. Statistically significant ORs are highlighted in bold

## Supplementary Materials

The following are available online at https://www.mdpi.com/2072-6643/12/6/1730/s1, Table S1. COVIDiet questionnaire used to collect information on dietary habits/behaviours and lifestyle factors during the COVID-19 Spanish confinement; Table S2. MEDAS-derived foods items by level of adherence to the MedDiet during the COVID-19 Spanish confinement; Table S3. Factors associated with adherence to the MedDiet during the Spanish confinement; Table S4. MEDAS-derived foods items by level of adherence to the MedDiet before the COVID-19 Spanish confinement; Table S5. Socio-demographic factors associated with the change in the adherence to the MedDiet during the Spanish confinement; Table S6. Subgroup analysis by gender on the association between fruits, vegetables and olive oil intake and the change in the adherence to the MedDiet during the COVID-19 Spanish confinement; Table S7. Sensitivity analyses performed on variables associated with the change in the adherence to the MedDiet during the Spanish confinement; Figure S1. Relationship between weeks and days of confinement with adherence to the MedDiet and the relative change of adherence to MedDiet; Figure S2. Google Trends output for Web search queries for different terms.
