# Supplementary material for: Changes in Dietary Behaviours during the COVID-19 Outbreak Confinement in the Spanish COVIDiet Study
Source: Nutrients. 2020 Jun 10;12(6):1730. doi: 10.3390/nu12061730 (PMC7353108; doi:10.3390/nu12061730)

Supplementary Material

Supplementary Table S1. COVIDiet questionnaire used to collect information on dietary habits/behaviours and lifestyle factors during the COVID-19 Spanish confinement. 2

Supplementary Table S2. MEDAS-derived foods items by level of adherence to the MedDiet during the COVID-19 Spanish confinement 6

Supplementary Table S3: Factors associated with adherence to the MedDiet during the Spanish confinement 7

Supplementary Table S4. MEDAS-derived foods items by level of adherence to the MedDiet before the COVID-19 Spanish confinement 9

Supplementary Table S5. Socio-demographic factors associated with the change in the adherence to the MedDiet during the Spanish confinement 11

Supplementary Table S6: Subgroup analysis by gender on the association between fruits, vegetables and olive oil intake and the change in the adherence to the MedDiet during the COVID-19 Spanish confinement 12

Supplementary Table S7: Sensitivity analyses performed on variables associated with the change in the adherence to the MedDiet during the Spanish confinement 13

Supplementary Figure S1: Relationship between weeks and days of confinement with adherence to the MedDiet and the relative change of adherence to MedDiet. 16

Supplementary Figure S2: Google Trends output for Web search queries for different terms. 17

Supplementary Table S1. COVIDiet questionnaire used to collect information on dietary habits/behaviours and lifestyle factors during the COVID-19 Spanish confinement.

**Socio-demographic information**
**Gender**
Male
Female
Other
**Place of residence**
Alone
Family home
Shared flat
Student's residence
**Country**
**Do you have children in care?**
Yes
No
**Education and training qualifications**
University degree
Postgraduate, Master degree, Doctorate
Professional and vocational training
Primary studies
None
Other
**Age**
Between 18 to 20 years old
Between 21 y 35 years old
Between 36 y 50 years old
Between 51 y 65 years old
More than de 65 years old
**Weight (kg)**
**Dietary habits related to healthy diet**
Please, answer the following questions based on the PREDIMED test. This test contains information about the adherence to the Mediterranean Diet which is considered as a reference of a healthy diet.
**Is olive oil your main source of added fat for cooking?**
Yes
No
**How much olive oil do you consume in total per day (including used for frying, meals out of the house, salads, etc)?**
4 or more tablespoons
2-3.9 tablespoons
0-1.9 tablespoons
**Are you using more olive oil during the confinement compared to your usual intake?**
Yes. My intake of olive oil is higher
No. My intake of olive oil is lower
My intake of olive oil remains as usual
**How many servings of vegetables do you consume per day? (the garnish or accompaniments = 1/2 serving) 1 serving = 200 g**
2 or more servings
1-1.9 servings
0-0.9 servings
**Have you increased the intake of vegetables during the confinement compared to your usual intake?**
Yes. My intake of vegetables is higher
No. My intake of vegetables is lower
My intake of vegetables remains as usual
**How many pieces of fruit (including fresh juice) do you consume per day?**
3 or more pieces
1-2.9 pieces
0-0.9 pieces
**Have you increased the intake of fruits during the confinement compared to your usual intake?**

Yes. My intake of fruits is higher
No. My intake of fruits is lower
My intake of olive fruits remains as usual
How many servings of red meats, hamburgers, sausages or deli meats do you consumer per day? (Servings: 100-150 g)
1 serving or more
0-0.9 servings
Have you increased the intake of red meats, hamburgers, sausages or deli meats during the confinement compared to your usual intake?
Yes. My intake of red meats, hamburgers, sausages or deli meats is higher
No. My intake of red meats, hamburgers, sausages or deli meats is lower
My intake of red meats, hamburgers, sausages or deli meats remains as usual
How many servings of butter, margarine or cream do you consume per day? (individual portion: 12 g)
1 serving or more
0-0.9 servings
How many carbonated and/or sugary beverages (soda, cola, tonic, bitter) do you consume per day?
1 serving or more
0-0.9 servings
Have you increased the intake of carbonated and/or sugary beverages (soda, cola, tonic, bitter) during the confinement compared to your usual intake?
Yes. My intake of carbonated and/or sugary beverages is higher
No. My intake of carbonated and/or sugary beverages is lower
My intake of carbonated and/or sugary beverages remains as usual
Do you drink wine? How much do you consume per week?
7 or more cups
3-6.9 cups
0-2.9 cups
I never drink wine
How many servings of pulses do you consume per week? (one serving dish: 150 g)
3 servings or more
1-2.9 servings
0-0.9 servings
Have you increased the intake of pulses during the confinement compared to your usual intake?
Yes. My intake of pulses is higher
No. My intake of pulses is lower
My intake of pulses remains as usual
How many servings of fish-seafood do you eat per week? 1 piece: 100-150 g of fish or 4-5 pieces or 200 g seafood)
3 servings or more
1-2.9 servings
0-0.9 servings
Have you increased the intake of fish-seafood during the confinement compared to your usual intake?
Yes. My intake of fish-seafood is higher
No. My intake of fish-seafood is lower
My intake of fish-seafood remains as usual
How often do you have commercial (non-homemade) pastries such as cookies, custards, sweets or cakes per week?
2 servings or more
0-1.9 servings
Have you increased the intake of commercial (non-homemade) pastries such as cookies, custards, sweets or cakes during the confinement compared to your usual intake?
Yes. My intake of commercial (non-homemade) pastries is higher
No. My intake of commercial (non-homemade) pastries is lower
My intake of commercial (non-homemade) pastries remains as usual
Have you increased the intake of homemade pastries such as cookies, custards, sweets or cakes during the confinement compared to your usual intake?
Yes. My intake of homemade pastries is higher
No. My intake of homemade pastries is lower
My intake of homemade pastries remains as usual

How often do you eat nuts per week?

3 servings or more

1-2.9 servings

0-0.9 servings

Do you prefer chicken, turkey or rabbit meat instead of veal, pork, hamburgers or sausages?

Yes

No

How many times a week do you consume cooked vegetables, pasta, rice or other dishes seasoned with tomato, garlic, onion or leek sauce made over low heat with olive oil (sofrito)

2 servings or more

1-1.9 servings

0-0.9 servings

Information about dietary habits

Due to the situation, it is possible that your dietary pattern has been altered. Thus, we request you to complete the additional items regarding to lifestyle, cooking way and intake pattern of some other food groups.

How many main dishes (breakfast, lunch or dinner) do you used to take daily out of home before the confinement?

1

2

3

None

Have you increased the intake of alcoholic beverages (wine, beer, high-grade drinks) during the confinement compared to your usual intake?

Yes. My intake of alcoholic beverages is higher

No. My intake of alcoholic beverages is lower

My intake of alcoholic beverages remains as usual

Have you found difficult finding any type of food?

Yes

No

In case you have found difficult finding any type of food, please, indicate which food

What type of cooking are you employing these days?

Fried

Oven

Microwave

Griddle

Stew

Are you cooking more often than before the confinement started?

Yes. I am cooking more now

No. I am cooking less now

I am cooking as usual

Have you increased the intake of fried foods?

Yes. My intake of fried foods is higher

No. My intake of fried foods is lower

My intake of fried foods remains as usual

During the confinement, how often are you consuming fried food?

Less than once time a week

1-3 times a week

4-6 times a week

7 or more than 7 times a week

Never

When you consume fried foods, what type of oil do you use?

Olive oil

Sunflower oil

Other

Have you increased the frequency of snacking during the confinement compared to your usual intake?

Yes. My snacking frequency is higher

No. My snacking frequency is lower

My snacking frequency remains as usual

Have you increased the intake of fast-food during the confinement compared to your usual intake?
Yes. My intake of fast-food is higher
No. My intake of fast-food is lower
My intake of fast-food remains as usual
Do you think that you are eating more than usual during the confinement?
Yes
No
Have you modified your physical activity?
It has increased
It has decreased
It remains as usual
I do not practice physical activity
Have you gained weight during the confinement?
Yes
No
I do not know

Supplementary Table S2. MEDAS-derived foods items by level of adherence to the MedDiet during the COVID-19 Spanish confinement

MEDAS food groups[1]		All (N=7,514)		Low (N=1,275)		Medium (N=4,129)		High (N=2,110)		p-value[2]
		N	%	N	%	N	%	N	%	
**1. Olive oil for cooking**										<0.001
	No	135	1.8	48	3.8	68	1.6	19	0.9	
	Yes	7379	98.2	1227	96.2	4061	98.4	2091	99.1	
**2. Olive oil (tablespoons/d)**										<0.001
	>4	2149	28.6	176	13.8	1035	25.1	938	44.5	
	0-1.9	1344	17.9	311	24.4	756	18.3	277	13.1	
	2-3.9	4021	53.5	788	61.8	2338	56.6	895	42.4	
**3. Vegetables (servings/d)**										<0.001
	>2	2559	34.1	17	1.3	937	22.7	1605	76.1	
	0-0.9	1276	17.0	504	39.5	712	17.2	60	2.8	
	1-1.9	3679	49.0	754	59.1	2480	60.1	445	21.1	
**4. Fruits (units/d)**										<0.001
	>3	1851	24.6	47	3.7	686	16.6	1118	53.0	
	0-0.9	1470	19.6	482	37.8	847	20.5	141	6.7	
	1-2.9	4193	55.8	746	58.5	2596	62.9	851	40.3	
**5. Red meat (servings/d)**										<0.001
	>1	1242	16.5	612	48.0	546	13.2	84	4.0	
	0-0.9	6272	83.5	663	52.0	3583	86.8	2026	96.0	
**6. Fats (servings/d)**										<0.001
	>1	351	4.7	159	12.5	171	4.1	21	1.0	
	0-0.9	7163	95.3	1116	87.5	3958	95.9	2089	99.0	
**7. Sweet beverages (servings/d)**										<0.001
	>1	722	9.6	378	29.6	301	7.3	43	2.0	
	0-0.9	6792	90.4	897	70.4	3828	92.7	2067	98.0	
**8. Wine (glasses/d)**										<0.001
	>7	232	3.1	5	0.4	108	2.6	119	5.6	
	0-2.9	2719	36.2	385	30.2	1527	37.0	807	38.2	
	3-6.9	677	9.0	100	7.8	361	8.7	216	10.2	
	Never	3886	51.7	785	61.6	2133	51.7	968	45.9	
**9. Legumes (servings/w)**										<0.001
	>3	1449	19.3	51	4.0	569	13.8	829	39.3	
	0-0.9	1209	16.1	354	27.8	676	16.4	179	8.5	
	1-2.9	4856	64.6	870	68.2	2884	69.8	1102	52.2	
**10. Fish (servings/w)**										<0.001
	>3	1241	16.5	26	2.0	473	11.5	742	35.2	
	0-0.9	2174	28.9	526	41.3	1263	30.6	385	18.2	
	1-2.9	4099	54.6	723	56.7	2393	58.0	983	46.6	
**11. Non-homemade pastries (units/w)**										<0.001
	>2	1832	24.4	748	58.7	910	22.0	174	8.2	
	0-1.9	5682	75.6	527	41.3	3219	78.0	1936	91.8	
**12. Nuts (servings/w)**										<0.001
	>3	2461	32.8	106	8.3	1049	25.4	1306	61.9	
	0-0.9	2822	37.6	687	53.9	1778	43.1	357	16.9	
	1-2.9	2231	29.7	482	37.8	1302	31.5	447	21.2	
**13. While meat preference**										<0.001
	No	1434	19.1	537	42.1	705	17.1	192	9.1	
	Yes	6080	80.9	738	57.9	3424	82.9	1918	90.9	
**14. Sofrito[3] (servings/w)**										<0.001
	>2	2603	34.6	147	11.5	1357	32.9	1099	52.1	
	0-0.9	1277	17.0	282	22.1	796	19.3	199	9.4	
	1-2.9	3634	48.4	846	66.4	1976	47.9	812	38.5	

[1] Foods groups derived from the 14-item MEDAS questionnaire as described in Table S1 (Supplementary material)
[2] Differences between the three MedDiet adherence groups were evaluated by the Chi-squared test.
[3] Sofrito is a mixture of lightly fried onions and garlic, usually with tomatoes and other vegetables, used as a base for soups and stews.

Supplementary Table S3: Factors associated with adherence to the MedDiet during the Spanish confinement

		High vs medium-low adherence to MedDiet					
		Crude Model		Model 1		Model 2	
		OR	95% CI	OR	95% CI	OR	95% CI
**Gender[1]**							
	Men	Ref.	Ref.				
	Women	1.19	[1.06;1.33]				
**Place of residence**							
	Family home	Ref.	Ref.	Ref.	Ref.		
	Shared flat	0.75	[0.61;0.92]	0.85	[0.68;1.06]		
	Student residence	0.27	[0.08;0.88]	0.3	[0.09;1]		
	Alone	0.93	[0.79;1.1]	0.93	[0.79;1.1]		
**Region by areas[2]**							
	Central	Ref.	Ref.				
	North	1.09	[0.93;1.26]				
	South	0.97	[0.86;1.09]				
**Children in care**							
	No	Ref.	Ref.	Ref.	Ref.	Ref.	Ref.
	Yes	1.05	[0.95;1.17]	0.94	[0.84;1.06]	0.93	[0.82;1.06]
**Educational level**							
	Basic	Ref.	Ref.	Ref.	Ref.		
	Professional	1.05	[0.82;1.35]	1.11	[0.86;1.43]		
	University	1.44	[1.17;1.78]	1.5	[1.21;1.85]		
	Postgraduate	1.59	[1.28;1.96]	1.59	[1.28;1.96]		
	Without	1.70	[0.71;4.13]	1.70	[0.71;4.13]		
**Age (years)**							
	21-35	Ref.	Ref.				
	36-50	1.2	[1.06;1.36]				
	51-65	1.35	[1.19;1.54]				
	>65	1.51	[1.21;1.88]				
	<20	0.86	[0.62;1.19]				
**Meals out-of-home**							
	0	Ref.	Ref.	Ref.	Ref.	Ref.	Ref.
	1	0.7	[0.62;0.79]	0.72	[0.64;0.82]	0.71	[0.63;0.81]
	2	0.53	[0.43;0.64]	0.55	[0.46;0.67]	0.56	[0.46;0.68]
	3	0.67	[0.59;0.77]	0.69	[0.61;0.79]	0.72	[0.62;0.83]
**Alcohol intake**							
	As before	Ref.	Ref.	Ref.	Ref.	Ref.	Ref.
	Lower	0.78	[0.7;0.86]	0.8	[0.71;0.89]	0.8	[0.71;0.89]
	Higher	0.61	[0.5;0.73]	0.62	[0.51;0.75]	0.61	[0.5;0.74]
**Difficult finding foods**							
	No	Ref.	Ref.	Ref.	Ref.	Ref.	Ref.
	Yes	0.97	[0.87;1.09]	1.02	[0.91;1.15]	1.02	[0.91;1.15]
**Type of cooking**							
	Stew	Ref.	Ref.	Ref.	Ref.	Ref.	Ref.
	Fried	0.36	[0.25;0.5]	0.38	[0.27;0.53]	0.39	[0.27;0.55]
	Oven	0.96	[0.83;1.11]	1.00	[0.86;1.16]	0.96	[0.83;1.12]
	Microwave	0.77	[0.57;1.03]	0.77	[0.57;1.03]	0.77	[0.57;1.05]
	Griddle	0.97	[0.86;1.09]	0.97	[0.86;1.09]	0.97	[0.86;1.09]
**Frequency of cooking**							
	As before	Ref.	Ref.	Ref.	Ref.	Ref.	Ref.
	Lower	0.55	[0.4;0.75]	0.59	[0.43;0.81]	0.59	[0.43;0.8]
	Higher	0.81	[0.73;0.9]	0.83	[0.75;0.92]	0.8	[0.71;0.89]
**Fried foods intake**							
	As before	Ref.	Ref.	Ref.	Ref.	Ref.	Ref.
	Lower	0.91	[0.8;1.03]	0.92	[0.81;1.04]	0.91	[0.8;1.03]

	Higher	0.29	[0.22;0.39]	0.31	[0.23;0.42]	0.32	[0.24;0.44]
**Fried foods frequency per week**							
	1-3	Ref.	Ref.	Ref.	Ref.	Ref.	Ref.
	4-6	0.7	[0.51;0.97]	0.71	[0.52;0.99]	0.75	[0.54;1.04]
	>7	1.08	[0.4;2.93]	1.1	[0.41;2.99]	1.4	[0.51;3.87]
	<1	1.78	[1.58;2.01]	1.78	[1.58;2.01]	1.75	[1.55;1.98]
	Never	2.53	[2.2;2.9]	2.53	[2.2;2.9]		
**Oil used for frying**							
	Olive oil	Ref.	Ref.	Ref.	Ref.	Ref.	Ref.
	Sunflower	0.51	[0.42;0.61]	0.53	[0.44;0.64]	0.56	[0.46;0.68]
	None	1.74	[1.53;1.97]	1.82	[1.6;2.06]	1.8	[1.58;2.05]
	Other	0.93	[0.52;1.65]	0.99	[0.61;1.85]	1.11	[0.62;1.99]
**Snacking frequency**							
	As before	Ref.	Ref.	Ref.	Ref.	Ref.	Ref.
	Lower	0.99	[0.86;1.14]	0.99	[0.86;1.15]	1.01	[0.88;1.17]
	Higher	0.63	[0.57;0.71]	0.66	[0.59;0.74]	0.67	[0.6;0.76]
**Fast food frequency**							
	As before	Ref.	Ref.	Ref.	Ref.	Ref.	Ref.
	Lower	0.73	[0.65;0.81]	0.75	[0.67;0.84]	0.74	[0.66;0.83]
	Higher	0.27	[0.19;0.37]	0.29	[0.21;0.4]	0.3	[0.22;0.42]
**Eating more**							
	No	Ref.	Ref.	Ref.	Ref.	Ref.	Ref.
	Yes	0.54	[0.49;0.61]	0.56	[0.5;0.62]	0.58	[0.51;0.65]
**Physical activity**							
	As before	Ref.	Ref.	Ref.	Ref.		
	Lower	0.83	[0.73;0.94]	0.81	[0.71;0.92]		
	Higher	0.91	[0.77;1.08]	0.96	[0.81;1.14]		
	As before	0.34	[0.25;0.45]	0.34	[0.25;0.45]		
**Weight gain**		0.66	[0.61;0.75]	0.68	[0.61;0.76]		
	No	Ref.	Ref.	Ref.	Ref.	Ref.	Ref.
	Unknown	0.66	[0.59;0.74]	1.01	[1;1.03]	0.7	[0.63;0.79]
	Yes	0.53	[0.44;0.63]	0.53	[0.45;0.63]	0.56	[0.47;0.67]
**Days**		1.01	[1;1.03]	1.01	[1;1.03]	1.02	[1;1.03]
**Week**[3]							
	1	Ref.	Ref.	Ref.	Ref.	Ref.	Ref.
	2	1.06	[0.93;1.2]	1.09	[0.95;1.24]	1.12	[0.98;1.28]
	3	1.03	[0.85;1.25]	1.05	[0.87;1.28]	1.1	[0.9;1.34]

[1] Numbers do not sum up because there were five respondents who reported another gender (data not shown).
[2] Spanish Autonomous regions were grouped into areas: see footnote Table 1.
[3] Weeks or days of confinement
Crude model: unadjusted for any variable
Adjusted models. Model 1: adjusted for gender (men, women, other), age groups (<20, 20-35y, 25-50y, 50-65y, >65y) and regions (Central, Northern and Southern). Model 2: Model 1 and educational level (primary, professional, university, postgraduate), residence (family home, shared flat, residence, alone), physical activity (similar, higher, lower, never).

Supplementary Table S4. MEDAS-derived foods items by level of adherence to the MedDiet before the COVID-19 Spanish confinement

MEDAS food groups[1]		Low (N=2,447)		Medium (N=3,773)		High (N=1,294)		p-value[2]
		N	%	N	%	N	%	
**1. Olive oil for cooking**								<0.001
	No	70	2.9	55	1.5	10	0.8	
	Yes	2377	97.1	3718	98.5	1284	99.2	
**2. Olive oil (tablespoons/d)**								<0.001
	>4	435	17.8	1095	29.0	619	47.8	
	0-1.9	548	22.4	633	16.8	163	12.6	
	2-3.9	1464	59.8	2045	54.2	512	39.6	
**3. Vegetables (servings/d)**								<0.001
	>2	308	12.6	1258	33.3	993	76.7	
	0-0.9	701	28.6	539	14.3	36	2.8	
	1-1.9	1438	58.8	1976	52.4	265	20.5	
**4. Fruits (servings/d)**								<0.001
	>3	287	11.7	853	22.6	711	54.9	
	0-0.9	712	29.1	677	17.9	81	6.3	
	1-2.9	1448	59.2	2243	59.4	502	38.8	
**5. Red meat (servings/d)**								<0.001
	>1	760	31.1	430	11.4	52	4.0	
	0-0.9	1687	68.9	3343	88.6	1242	96.0	
**6. Fats (servings/d)**								<0.001
	>1	232	9.5	108	2.9	11	0.9	
	0-0.9	2215	90.5	3665	97.1	1283	99.1	
**7. Sweet beverages (servings/d)**								<0.001
	>1	474	19.4	228	6.	20	1.5	
	0-0.9	1973	80.6	3545	94.0	1274	98.5	
**8. Wine (glasses/d)**								<0.001
	>7	29	1.2	118	3.1	85	6.6	
	0-2.9	809	33.1	1415	37.5	495	38.3	
	3-6.9	196	8.0	343	9.1	138	10.7	
	Never	1413	57.7	1897	50.3	576	44.5	
**9. Legumes (servings/w)**								<0.001
	>3	221	9.0	707	18.7	521	40.3	
	0-0.9	537	21.9	563	14.9	109	8.4	
	1-2.9	1689	69.0	2503	66.3	664	51.3	
**10. Fish (servings/w)**								<0.001
	>3	151	6.2	593	15.7	497	38.4	
	0-0.9	938	38.3	1016	26.9	220	17.0	
	1-2.9	1358	55.5	2164	57.4	577	44.6	
**11. Non-homemade pastries (unit/day)**								<0.001
	>2	1005	41.1	727	19.3	100	7.7	
	0-1.9	1442	58.9	3046	80.7	1194	92.3	
**12. Nuts (servings/w)**								<0.001
	>3	330	13.5	1264	33.5	867	67.0	
	0-0.9	1227	50.1	1420	37.6	175	13.5	
	1-2.9	890	36.4	1089	28.9	252	19.5	
**13. White meat**								<0.001
	No	773	31.6	546	14.5	115	8.9	
	Yes	1674	68.40	3227	85.50	1179	91.1	
**14. Sofrito[3] (servings/w)**								<0.001
	>3	462	18.9	1463	38.8	678	52.4	
	0-0.9	509	20.8	652	17.3	116	9.0	
	1-2.9	1476	60.3	1658	43.9	500	38.6	

[1] Foods groups derived from the 14-item MEDAS questionnaire as described in Table S1 (Supplementary material)

[2] Differences between the three adherence groups in were evaluated by the Chi-squared test.
[3]Sofrito is a mixture of lightly fried onions and garlic, usually with tomatoes and other vegetables, used as a base for soups and stews.

Supplementary Table S5. Socio-demographic factors associated with the change in the adherence to the MedDiet during the Spanish confinement

	Non-change N=4,122		Change in MD adherence N=3,392		p-value[2]	Crude model[3]		Adjusted model[4]	
	**N**	**%**	**N**	**%**		**OR**	**95% CI**	**OR**	**95% CI**
**Gender[1]**					0.391				
Men	1235	30.0	969	28.6		Ref.		NA	
Women	2884	70.0	2421	71.4		1.07	[0.97;1.18]		
**Place of residence**					<0.001				
Family home	3440	83.5	2710	79.9		Ref.		Ref.	
Shared flat	282	6.8	253	7.5		1.14	[0.95;1.36]	1.1	[0.91;1.33]
Student residence	13	0.3	18	0.5		1.75	[0.86;3.68]	1.69	[0.82;3.46]
Alone	387	9.4	411	12.1		**1.35**	**[1.16;1.56]**	**1.36**	**[1.17;1.58]**
**Region by areas[5]**					0.028				
Central	1205	29.2	1061	31.3		Ref.		Ref.	
North	708	17.2	515	15.2		**0.83**	**[0.72;0.95]**	**0.67**	**[0.46;0.95]**
South	2209	53.6	1816	53.5		0.93	[0.84;1.04]	0.92	[0.67;1.26]
**Children in care**					0.024				
No	2422	58.8	2081	61.4		Ref.		Ref.	
Yes	1700	41.2	1311	38.6		**0.90**	**[0.82;0.98]**	**0.90**	**[0.81;1.00]**
**Educational level**					0.010				
University	1941	47.1	1546	45.6		Ref.		Ref.	
Postgraduate	1237	30.0	1133	33.4		**1.15**	**[1.04;1.28]**	**1.13**	**[1.02;1.26]**
Professional	586	14.2	456	13.4		0.98	[0.85;1.12]	0.98	[0.85;1.12]
Primary	358	8.7	257	7.6		0.90	[0.76;1.07]	0.91	[0.76;1.09]
**Age (years)**					0.008				
21-35	1378	33.4	1180	34.8		Ref.		NA	
36-50	1279	31.0	1092	32.2		1.00	[0.89;1.12]		
51-65	1100	26.7	828	24.4		**0.88**	**[0.78;0.99]**		
>65	255	6.2	173	5.1		**0.79**	**[0.64;0.98]**		
<20	110	2.7	119	3.5		1.26	[0.96;1.66]		
	**Mean**	**SD**	**Mean**	**SD**	**p-value**	**OR**	**95% CI**	**OR**	**95% CI**
Days	10.1	3.30	10.2	3.37	0.144	1.01	[1.00;1.02]	1.02	**[1.00;1.03]**
Weight	68.5	17.6	69.2	14.6	0.073	1.00	[1.00;1.01]	**1.01**	**[1.00;1.01]**

[1] Numbers do not sum up because there were five respondents who reported another gender (data not shown).
[2] Differences between the groups were evaluated by the Chi-squared test.
[3] Crude model: unadjusted for any variable
[4] Adjusted model: adjusted for gender (men, women, other), age groups (<20y, 20-35y, 25-50y, 50-65y, >65y), regions (Central, Northern and Southern), educational level (primary, professional, university, postgraduate), residence (family home, shared flat, residence, alone), physical activity (similar, higher, lower, never). For every variable, the adjusting variable was simultaneously removed from the model.
[5] Spanish Autonomous regions were grouped into areas: see Table 1 footnote.
Statistically significant ORs are highlighted in bold.

Supplementary Table S6: Subgroup analysis by gender on the association between fruits, vegetables and olive oil intake and the change in the adherence to the MedDiet during the COVID-19 Spanish confinement

		Men (N=2,204)		Women (N=5,305)	
		**OR[1]**	**95% CI**	**OR[1]**	**95% CI**
**Olive oil (tablespoons/d)**					
	As before	Ref.	Ref.	Ref.	Ref.
	Lower	3.13	[2.20;4.44]	3.13	[2.45;4.05]
	Higher	8.00	[5.40;12.32]	3.71	[2.94;4.65]
	p-value for interaction by gender: 0.006				
**Vegetables (servings/d)**					
	As before	Ref.	Ref.	Ref.	Ref.
	Lower	2.32	[1.76;3.06]	1.34	[1.12;1.59]
	Higher	7.17	[5.43;9.57]	8.25	[6.81;10.06]
	p-value for interaction by gender: 0.001				
**Fruits (servings/d)**					
	As before	Ref.	Ref.	Ref.	Ref.
	Lower	2.89	[2.18;3.83]	1.82	[1.55;2.14]
	Higher	6.30	[4.90;8.16]	6.05	[5.10;7.18]
	p-value for interaction by gender: 0.016				

[1] Adjusted model: adjusted for age groups (<20, 20-35y, 25-50y, 50-65y, >65y), regions (Central, Northern and Southern), educational level (primary, professional, university, postgraduate), residence (family home, shared flat, residence, alone), physical activity (similar, higher, lower, never).

Supplementary Table S7: Sensitivity analyses performed on variables associated with the change in the adherence to the MedDiet during the Spanish confinement

	1. Removing first week of confinement							2. As relative change (high > vs low)							3. Improved MedDiet[5]	
	Non-change		Change in MD adherence					Non-change		Change in MD adherence					Non-change vs change	
	N=3,297		N=2,702			Crude Model		N=4,873		N=2,641		p-value[3]	Crude Model		Crude Model	
	N	%	N	%	p-value[3]	OR	95% CI	N	%	N	%		OR	95% CI	OR	95% CI
**Gender[1]**					0.796							0.902				
men	935	28.4	747	27.6		Ref.	Ref.	1422	29.2	782	29.6		Ref.	Ref.	Ref.	Ref.
women	2359	71.5	1958	72.3		1.04	[0.93;1.16]	3448	70.8	1857	70.3		0.98	[0.88;1.09]	1.09	[0.98;1.2]
**Place of residence**					<0.001							<0.001				
Family home	2739	83.1	2146	79.3		Ref.	Ref.	4060	83.3	2090	79.1		Ref.	Ref.	Ref.	Ref.
Shared flat	244	7.40	214	7.91		1.12	[0.92;1.36]	326	6.69	209	7.91		1.25	[1.04;1.49]	1.11	[0.93;1.33]
Residence	10	0.30	13	0.48		1.65	[0.72;3.92]	16	0.33	15	0.57		1.82	[0.88;3.73]	1.48	[0.72;3.02]
Alone	304	9.22	334	12.3		1.40	[1.19;1.65]	471	9.67	327	12.4		1.35	[1.16;1.57]	1.31	[1.13;1.52]
**Region by areas[2]**					0.019							0.022				
Central	1017	30.8	904	33.4		Ref.	Ref.	1439	29.5	827	31.3		Ref.	Ref.	Ref.	Ref.
North	670	20.3	482	17.8		0.81	[0.70;0.94]	833	17.1	390	14.8		0.81	[0.70;0.94]	0.86	[0.75;0.99]
South	1610	48.8	1321	48.8		0.92	[0.82;1.04]	2601	53.4	1424	53.9		0.95	[0.86;1.06]	0.97	[0.87;1.07]
**Children in care**					0.019							0.002				
No	1968	59.7	1697	62.7		Ref.	Ref.	2858	58.6	1645	62.3		Ref.	Ref.	Ref.	Ref.
Yes	1329	40.3	1010	37.3		0.88	[0.79;0.98]	2015	41.4	996	37.7		0.86	[0.78;0.95]	0.87	[0.8;0.96]
**Educational level**					0.015							0.514				
Basic	293	8.89	206	7.61		Ref.	Ref.	395	8.11	198	7.50		Ref.	Ref.	Ref.	Ref.
Professional	417	12.6	320	1.8		1.09	[0.87;1.37]	579	11.9	310	11.7		1.07	[0.86;1.33]	1.13	[0.92;1.39]
University	1502	45.6	1198	44.3		1.13	[0.93;1.38]	2279	46.8	1208	45.7		1.06	[0.88;1.27]	1.1	[0.93;1.31]
Postgraduate	997	30.2	925	34.2		1.32	[1.08;1.61]	1506	30.9	864	32.7		1.14	[0.95;1.39]	1.19	[0.99;1.43]
Without	9	0.27	9	0.33		1.42	[0.54;3.75]	12	0.25	10	0.38		1.67	[0.68;3.96]	1.68	[0.69;4.03]
**Age (years)**					0.014							0.009				
21-35	1186	36.0	1011	37.3		Ref.	Ref.	1621	33.3	937	35.5		Ref.	Ref.	Ref.	Ref.
36-50	1044	31.7	893	33.0		1.00	[0.89;1.13]	1533	31.5	838	31.7		0.95	[0.84;1.06]	0.96	[0.86;1.08]
51-65	780	23.7	571	21.1		0.86	[0.75;0.98]	1294	26.6	634	24.0		0.85	[0.75;0.96]	0.79	[0.7;0.89]
>65	191	5.79	129	4.77		0.79	[0.62;1.01]	292	5.99	136	5.15		0.81	[0.65;1.00]	0.69	[0.57;0.85]
<20	96	2.91	103	3.80		1.26	[0.94;1.68]	133	2.73	96	3.63		1.25	[0.95;1.64]	1.61	[1.22;2.12]
**Meals out-of-home**					<0.001							<0.001				

0	1387	42.1	935	34.5		Ref.	Ref.	2093	43.0	882	33.4		Ref.	Ref.	Ref.	Ref.
1	968	29.4	811	30.0		1.24	[1.10;1.41]	1416	29.1	791	30.0		1.33	[1.18;1.49]	1.24	[1.11;1.39]
2	266	8.07	327	12.1		1.82	[1.52;2.19]	411	8.43	347	13.1		2.00	[1.70;2.36]	1.83	[1.56;2.15]
3	676	20.5	634	23.4		1.39	[1.21;1.59]	953	19.6	621	23.5		1.55	[1.36;1.76]	1.38	[1.22;1.56]
**Alcohol intake**					<0.001							<0.001				
As before	1260	38.2	622	23.0		Ref.	Ref.	1865	38.3	564	21.4		Ref.	Ref.	Ref.	Ref.
Lower	1675	50.8	1811	66.9		2.19	[1.95;2.46]	2481	50.9	1821	69.0		2.43	[2.17;2.72]	2.29	[2.07;2.54]
Higher	362	11.0	274	10.1		1.53	[1.27;1.84]	527	10.8	256	9.69		1.61	[1.35;1.92]	1.62	[1.38;1.91]
**Difficult finding foods**					<0.001							0.001				
No	2454	74.4	1889	69.8		Ref.	Ref.	3610	74.1	1858	70.4		Ref.	Ref.	Ref.	Ref.
Yes	843	25.6	818	30.2		1.26	[1.13;1.41]	1263	25.9	783	29.6		1.20	[1.08;1.34]	1.26	[1.13;1.39]
**Type of cooking**					0.224							0.843				
Stew	938	28.5	781	28.9		Ref.	Ref.	1440	29.6	768	29.1		Ref.	Ref.	Ref.	Ref.
Fried	152	4.61	102	3.77		0.81	[0.62;1.05]	205	4.21	105	3.98		0.96	[0.75;1.23]	0.96	[0.85;1.20]
Oven	611	18.5	546	20.2		1.07	[0.92;1.25]	892	18.3	506	19.2		1.06	[0.92;1.22]	1.1	[0.97;1.26]
Microwave	118	3.58	85	3.14		0.87	[0.64;1.16]	174	3.57	87	3.29		0.94	[0.71;1.23]	1.02	[0.79;1.32]
Griddle	1478	44.8	1193	44.1		0.97	[0.86;1.10]	2162	44.4	1175	44.5		1.02	[0.91;1.14]	1.06	[0.95;1.18]
**Frequency of cooking**					<0.001							<0.001				
As before	1871	56.7	1126	41.6		Ref.	Ref.	2735	56.1	1074	40.7		Ref.	Ref.	Ref.	Ref.
Lower	98	2.97	125	4.62		2.12	[1.61;2.79]	144	2.96	129	4.88		2.28	[1.78;2.92]	2.61	[2.02;3.38]
Higher	1328	40.3	1456	53.8		1.82	[1.64;2.02]	1994	40.9	1438	54.4		1.84	[1.67;2.03]	1.87	[1.7;2.05]
**Fried foods intake**					<0.001							<0.001				
As before	2749	83.4	1677	62.0		Ref.	Ref.	4015	82.4	1502	56.9		Ref.	Ref.	Ref.	Ref.
Lower	320	9.71	872	32.2		4.46	[3.88;5.15]	558	11.5	967	36.6		4.63	[4.11;5.22]	4.59	[4.04;5.21]
Higher	228	6.92	158	5.84		1.14	[0.92;1.40]	300	6.16	172	6.51		1.53	[1.26;1.86]	1.45	[1.26;1.86]
**Fried foods frequency per week**					0.007							0.268				
1-3	1321	40.1	978	36.1		Ref.	Ref.	1905	39.1	989	37.4		Ref.	Ref.	Ref.	Ref.
4-6	145	4.40	108	3.99		1.01	[0.77;1.31]	201	4.12	108	4.09		1.04	[0.81;1.32]	1.22	[0.96;1.54]
>7	11	0.33	9	0.33		1.11	[0.44;2.72]	17	0.35	6	0.23		0.69	[0.25;1.68]	1.12	[0.49;2.54]
<1	1184	35.9	1006	37.2		1.15	[1.02;1.29]	1789	36.7	966	36.6		1.04	[0.93;1.16]	0.95	[0.85;1.05]
Never	636	19.3	606	22.4		1.29	[1.12;1.48]	961	19.7	572	21.7		1.15	[1.01;1.30]	1.04	[0.92;1.18]
**Oil used for frying**					0.137							0.070				
Olive oil	2249	68.2	1779	65.7		Ref.	Ref.	3375	69.3	1764	66.8		Ref.	Ref.	Ref.	Ref.
Sunflower	415	12.6	349	12.9		1.06	[0.91;1.24]	571	11.7	359	13.6		1.20	[1.04;1.39]	1.2	[1.04;1.38]
None	611	18.5	563	20.8		1.16	[1.02;1.33]	885	18.2	498	18.9		1.08	[0.95;1.22]	1.04	[0.93;1.17]

Other	22	0.67	16	0.59		0.92	[0.47;1.76]	42	0.86	20	0.76		0.91	[0.52;1.55]	0.99	[0.6;1.64]
**Snacking frequency**					<0.001							<0.001				
As before	1729	52.4	1072	39.6		Ref.	Ref.	2527	51.9	984	37.3		Ref.	Ref.	Ref.	Ref.
Lower	296	8.98	636	23.5		3.46	[2.96;4.06]	488	10.0	688	26.1		3.62	[3.16;4.15]	5.69	[4.88;6.65]
Higher	1272	38.6	999	36.9		1.27	[1.13;1.42]	1858	38.1	969	36.7		1.34	[1.20;1.49]	1.56	[1.41;1.72]
**Fast food frequency**					<0.001							<0.001				
As before	2290	69.5	1258	46.5		Ref.	Ref.	3400	69.8	1107	41.9		Ref.	Ref.	Ref.	Ref.
Lower	817	24.8	1327	49.0		2.96	[2.65;3.30]	1217	25.0	1406	53.2		3.55	[3.20;3.93]	3.03	[2.74;3.35]
Higher	190	5.76	122	4.51		1.17	[0.92;1.48]	256	5.25	128	4.85		1.54	[1.23;1.92]	2.01	[1.63;2.48]
**Eating more**					0.119							0.629				
No	2060	62.5	1745	64.5		Ref.	Ref.	3112	63.9	1671	63.3		Ref.	Ref.	Ref.	Ref.
Yes	1237	37.5	962	35.5		0.92	[0.83;1.02]	1761	36.1	970	36.7		1.03	[0.93;1.13]	1.1	[1;1.21]
**Physical activity**					0.057							0.101				
As before	614	18.6	518	19.1		Ref.	Ref.	922	18.9	481	18.2		Ref.	Ref.	Ref.	Ref.
Lower	1917	58.1	1566	57.9		0.97	[0.85;1.11]	2916	59.8	1559	59.0		1.02	[0.90;1.16]	1.03	[0.92;1.17]
Never	541	16.4	480	17.7		1.05	[0.89;1.25]	740	15.2	456	17.3		1.18	[1.01;1.39]	1.16	[0.99;1.35]
Never	225	6.82	143	5.28		0.75	[0.59;0.96]	295	6.05	145	5.49		0.94	[0.75;1.18]	0.85	[0.69;1.06]
**Weight gain**					0.195							0.373				
No	1531	46.4	1320	48.8		Ref.	Ref.	2299	47.2	1257	47.6		Ref.	Ref.	Ref.	Ref.
Unknown	1320	40.0	1032	38.1		0.91	[0.81;1.01]	1964	40.3	1029	39.0		0.96	[0.87;1.06]	0.96	[0.87;1.06]
Yes	446	13.5	355	13.1		0.92	[0.79;1.08]	610	12.5	355	13.4		1.06	[0.92;1.23]	1.2	[1.04;1.39]
**Week[4]**					0.050							0.292				
1	NA	NA	NA	NA				965	19.8	545	20.6		Ref.	Ref.	Ref.	Ref.
2	2864	86.9	2303	85.1		Ref.	Ref.	3380	69.4	1787	67.7		0.94	[0.83;1.06]	0.99	[0.89;1.11]
3	433	13.1	404	14.9		1.16	[1.00;1.34]	528	10.8	309	11.7		1.04	[0.87;1.23]	1.22	[1.03;1.45]
**Days**	10.9	3.26	11.0	3.32	0.078	1.01	[1.00;1.03]	10.1	3.31	10.1	3.36	0.143	1.00	[0.99;1.02]	1.02	[1;1.03]

[1] Numbers do not sum up because there were five respondents who reported another gender (data not shown).
[2] Spanish Autonomous regions were grouped into areas: see footnote Table 1.
[3] Differences between the three MedDiet adherence groups were evaluated by the Chi-squared test.
[4] Weeks or days of confinement
[5] Improved MedDiet score refers to MedDiet before the confinement by accounting for snaking, alcohol consumption and intake of fried food in the MEDAS scoring. Change in adherence to MedDiet was calculated as described in the methods section. Non-change (N=3,808) vs change (N=3,706) in adherence to the MedDiet was likewise estimated.
Crude model: unadjusted for any variable.

Supplementary Figure S1: Relationship between weeks and days of confinement with adherence to the MedDiet and the relative change of adherence to MedDiet.

Figure caption: First column shows splines (multivariate-adjusted) of ORs of adherence to the MedDiet by days of confinement (top **- A**) and ORs of change in adherence to the MedDiet by days of confinement (bottom **-C**). Second column shows boxplots of adherence to the MedDiet, before and during confinement, and relative change of adherence to the MedDiet by weeks of confinement (**B**). Differences in mean adherence by groups were evaluated by means of the Kruskal-Wallis test.

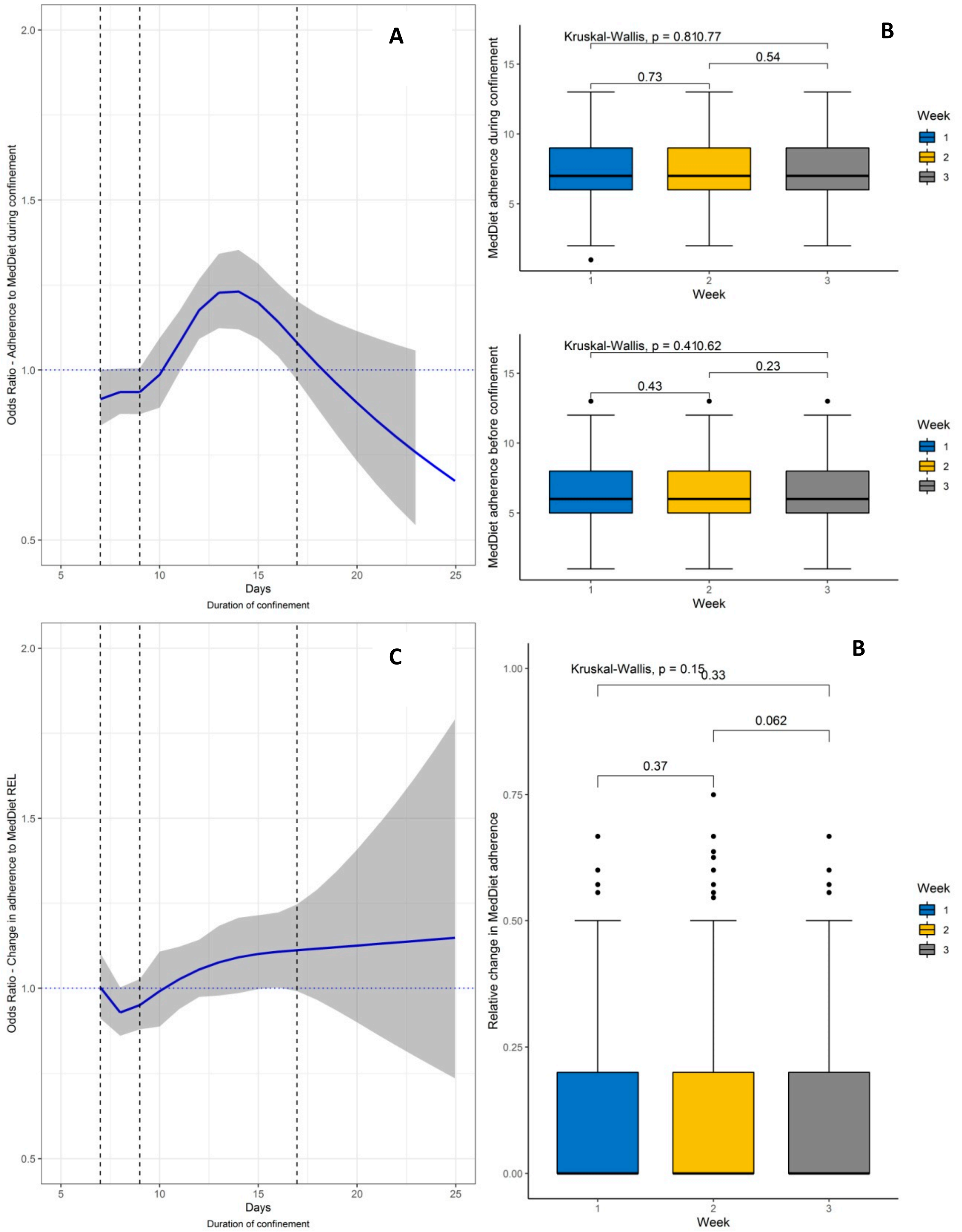

Supplementary Figure S2: Google Trends output for Web search queries for different terms.

Figure caption: Google Trends output for Web search queries for the term (a) "Bizcocho casero" (Homemade cake); (b) "Recetas caseras" (Traditional recipes) and (c) "Entrenamiento en casa" (Home workout) in Spain from March 2014 to March 2020 (left) and from February, 22nd to March 17th (right).

(a)

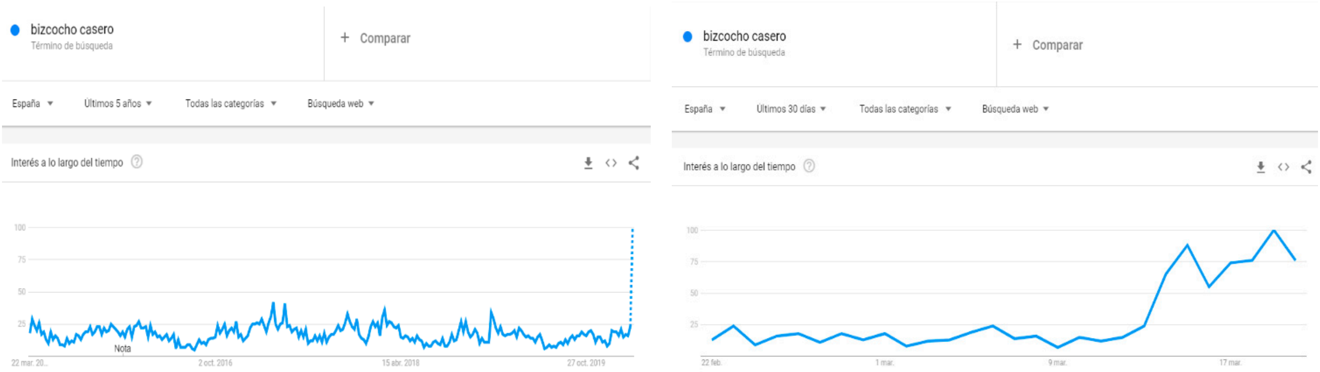

(b)

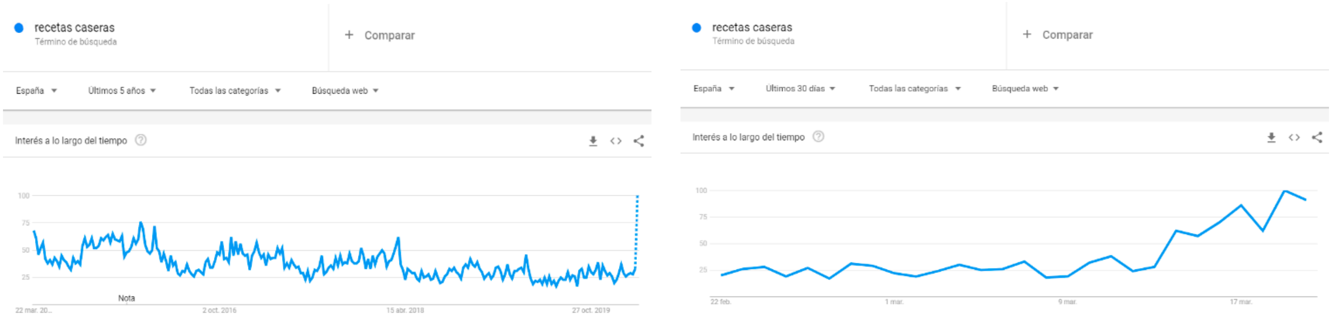

(c)

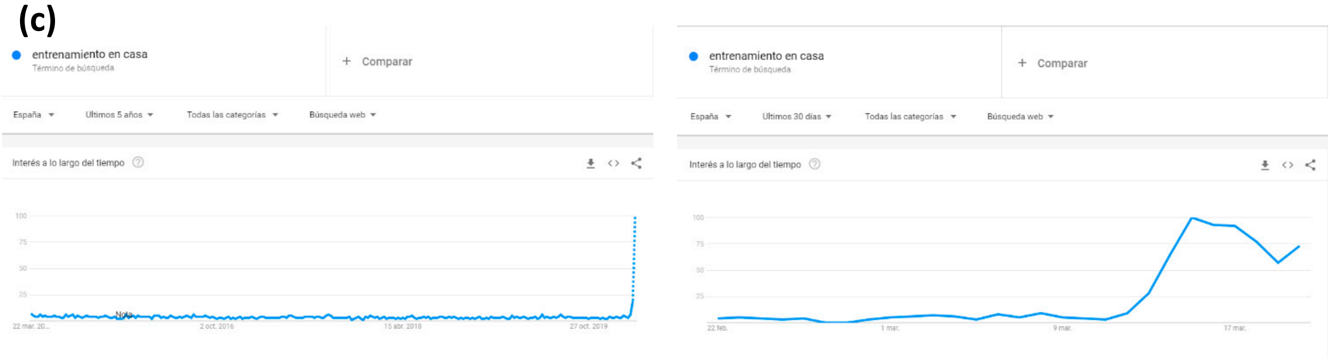

Supplement: Supplementary file 1 [file nutrients-12-01730-s001.pdf]
